# Picosecond Bessel Beam Fabricated Pure, Gold-Coated Silver Nanostructures for Trace-Level Sensing of Multiple Explosives and Hazardous Molecules

**DOI:** 10.3390/ma15124155

**Published:** 2022-06-11

**Authors:** Dipanjan Banerjee, Mangababu Akkanaboina, Subhasree Ghosh, Venugopal Rao Soma

**Affiliations:** 1 Advanced Centre of Research in High Energy Materials (ACRHEM), University of Hyderabad, Hyderabad 500046, Telangana, India; 19acpp02@uohyd.ac.in; 2 School of Physics, University of Hyderabad, Hyderabad 50006, Telangana, India; 17phph19@uohyd.ac.in; 3 Department of Biotechnology and Bioinformatics, School of Life Sciences, University of Hyderabad, Hyderabad 500046, Telangana, India; 17ltph17@uohyd.ac.in

**Keywords:** picosecond ablation, Bessel beam, SERS, silver nanostructures, picric acid, thiram

## Abstract

A zeroth-order, non-diffracting Bessel beam, generated by picosecond laser pulses (1064 nm, 10 Hz, 30 ps) through an axicon, was utilized to perform pulse energy-dependent (12 mJ, 16 mJ, 20 mJ, 24 mJ) laser ablation of silver (Ag) substrates in air. The fabrication resulted in finger-like Ag nanostructures (NSs) in the sub-200 nm domain and obtained structures were characterized using the FESEM and AFM techniques. Subsequently, we employed those Ag NSs in surface-enhanced Raman spectroscopy (SERS) studies achieving promising sensing results towards trace-level detection of six different hazardous materials (explosive molecules of picric acid (PA) and ammonium nitrate (AN), a pesticide thiram (TH) and the dye molecules of Methylene Blue (MB), Malachite Green (MG), and Nile Blue (NB)) along with a biomolecule (hen egg white lysozyme (HEWL)). The remarkably superior plasmonic behaviour exhibited by the AgNS corresponding to 16 mJ pulse ablation energy was further explored. To accomplish a real-time application-oriented understanding, time-dependent studies were performed utilizing the AgNS prepared with 16 mJ and TH molecule by collecting the SERS data periodically for up to 120 days. The coated AgNSs were prepared with optimized gold (Au) deposition, accomplishing a much lower trace detection in the case of thiram (~50 pM compared to ~50 nM achieved prior to the coating) as well as superior EF up to ~10^8^ (~10^6^ before Au coating). Additionally, these substrates have demonstrated superior stability compared to those obtained before Au coating.

## 1. Introduction

Globally, fascinating advancements have been realized so far in the realm of laser–matter interaction and nano-photonics. Scientists have unveiled, and in some cases pioneered, intriguing aspects of the nano-world involving the interplay of ultrafast pulsed lasers. Across the scientific community, numerous innovative approaches have been pursued in order to reach a high-flourishing upswing in optical responses of the nano-photonic systems in diverse terms. Aiming for such advancements in miscellaneous nano-scale optical features, such as fabrication of diverse nanoparticles (NPs) in liquids [1,2,3,4,5], exotic nanostructures (NSs) (in air/liquid) with desired plasmonic response, and attaining enhanced optical features, such as surface-enhanced Raman spectroscopy (SERS), significant improvisations have been undertaken in laser–matter interaction research. Implementation of non-Gaussian beam profile in laser ablation, and engagement of different off-axis geometrical optics has been quite significant. As in the case of a laser–matter interaction, ablation energy deposition is a crucial aspect, generation and implementation of the transformed beam profile also originated from the same fundamental motivation. In this regard, non-diffracting beams have offered additional advantages in terms. of higher depth of focus, invariance of core-intensity profile with propagation, etc., making them a fascinating and potential candidate in the domain of laser ablation. Among interesting non-diffracting beams [6], we have a number of varieties, from an elliptical family of non-diffracting Mathieu beams, described by Mathieu functions to non-diffracting plane waves in Cartesian coordinates and also parabolic beams found by Bandres et al. [7]. Several other structured beams [8,9,10,11,12,13] have also been generated and used for surface morphology/roughness modification studies. Bessel beam (BB) is one of the most significant among various non-diffracting/structured beams. Several methods have been surveyed to produce a BB. Computer-generated holograms have been combined with an axicon using a spatial light modulator (SLM) to generate an achromatic BB. SLM, along with an interactive Fourier transform algorithm, was also reported. Likewise, arrays of BB have been generated by Stoyanov et al. [14]. The aforementioned advantages, such as immunity to diffraction and invariance to transverse profile, make BB an attractive alternative for various optical applications. Higher-order BBs have also been achieved utilizing axicon lenses by Arlt et al. [15].

BB has been found to accomplish extensive and exciting photonic applications in various modern age fields, such as materials processing [16,17,18,19,20,21,22,23,24,25,26], photopolymerization [27], and microactuator fabrication [28]. BBs have also been extensively implemented for particle trapping [29], particle handling applications [30], or acoustic radiation force strategies in liquids [31]. Stankevičius et al. [32] fabricated concentric mirroring polymeric structures containing gold nanoparticles by employing a bb-like beam array with 30 μm and 60 μm periods. Later they utilized those for surface-enhanced Raman spectroscopy (SERS) sensing purposes. Extensive investigations on c-Si, forming trench/ripple-like structures with polarization and varied, focused fs BB can be found in the report of Sugawara et al. [33]. Interesting results have been revealed in the studies of Kumar et al. [34], wherein they demonstrated surface machining on the monocrystalline synthetic diamond with a pulsed BB. Along with that, a systematic comparative study has also been presented on sapphire crystals with varying pulse energy, pulse width, and axicon cone angle. Odachi et al. [35] presented fascinating pre-ablation results, utilizing a 786 nm femtosecond (fs) laser beam and its second harmonic at 393 nm, employing a dual axicon combination on c-Si in a vacuum chamber. They have thoroughly investigated the laser-modified zone associated with low- and high-frequency periodic ripples [35]. Inoue et al. [36] have also carried out their micro-ablation investigations on crystalline Si, thoroughly characterizing the concentric-rings effect on the material processing, separately focusing on predicting the ablation-hole size. Tailored BB studies have also been reported by He et al. [37], wherein investigations pertaining to the fabrication of 2D arrays of Si-ablated forms were presented, leading to the bright applications of 3D ICs (integrated circuits). In the case of metal thin films, Sahin et al. [38] have been quite successful in fabricating nanoslit arrays, also indicating a potential application in enhanced sensing due to a shift in the resonance wavelength. Yalizay et al. [39] have also performed fs BB ablation of metal thin films, demonstrating excellent results regarding the ablating beam focal spot-size insensitivity towards longitudinal propagation. Notwithstanding the numerous studies reported with the BB, the pulsed laser–matter interaction of axicon-employed BB with plasmonic metals is lacking. Extraordinary possibilities in the sensing applications of BB interaction-fabricated plasmonic NSs, such as silver and gold, are hardly explored. Our group [40] has previously investigated BB ablation in liquids while focusing on the fabrication of NPs (colloids) and, significantly, those studies were performed in water and acetone. A few of our earlier studies [41,42,43,44,45,46] focused on the femtosecond (fs) laser ablation of bulk targets of Ag/Au (in ambient air as well as solvents) and Cu (in different solvents) while accomplishing hierarchal/diverse nanostructures (NSs), along with plasmonic NPs which were subsequently utilized for the sensing of explosive molecules successfully. However, the ablation in those cases was performed with fs pulses and a Gaussian input beam profile. To make a significant contribution in this completely unchallenged area of BB ambient ablation, we performed extensive research on energy-dependent fabrication of BB-induced silver (Ag) NSs using picosecond (ps) laser pulses at 1064 nm. The motivation of employing a Bessel beam was to utilize the additional advantages of higher depth of focus and invariance of the core-intensity profile with propagation, i.e., the strong resistance of its central maxima towards diffractive spreading along with spatial propagation [40]. Bessel beam ablation of silver in air has not been accomplished earlier and, we believe, this is one of the novelty aspects of this work. We have accomplished (a) complete characterization of the obtained exotic NSs using field emission scanning electron microscopy (FESEM) and AFM studies, (b) wide-ranging sensing data involving the Ag NSs through SERS studies for applications including real-time hazardous molecules detection (e.g., explosives, pesticides, biomolecules, and dyes), (c) superior SERS factors through a combination of Au coating on the best AuNS obtained (at 16 mJ), and (d) the demonstration of versatility, reproducibility, recyclability, and long-term stability of these substrates.

## 2. Materials and Methods

**Figure 1:**
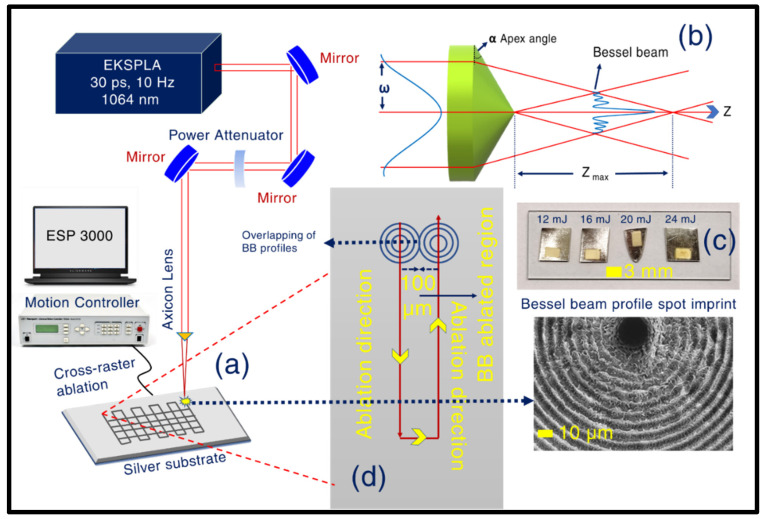
(**a**) Schematic of the experiment involving BB ablation in the air on Ag. The cross-raster scanning employed is also depicted in the figure with BB profile imprint observed at the initial ablation spot (indicated by the dashed line and inset), (**b**) Axicon prism lens and the generated BB, (**c**) pictures of the fabricated silver NSs with different input pulse energies (12 mJ, 16 mJ, 20 mJ, and 24 mJ), (**d**) Schematic of overlapping two BB profiles during the first raster scan.

Laser ablation in the air (LAA) was performed by engaging a focused 1064 nm beam from a 10 Hz, 30 ps laser (M/s EKSPLA, Lithuania), through an axicon (10^0^, IR range, AR coated, M/s Thorlabs, AX1210-B, Ely, UK) on the surface of the thoroughly cleaned silver substrate. The targets were purchased locally with >99.9% purity. The targets were cleaned by submerging them in ethanol, deionized water, and ethanol in sequence inside an ultrasonic cleaner for ~15 min to remove any possible surface impurities. The schematic of the experimental setup is presented in Figure 1a. Figure 1b depicts the focusing mechanism of the BB. Figure 1c shows the pictures of the actual Ag targets post ablation with the laser-irradiated regions clearly visible while Figure 1d illustrates the scanning mechanism used. A cross-raster scan was performed with the focused beam ablating the plasmonic substrate over an area of (4 × 3 mm^2^). Two X-Y stages, controlled with ESP 3000 motion controller (Newport, OR, USA), were used to perform the cross-raster scan with an ESP 3000 user interface-based program. The scan speed was set at 0.5 mm/s, along with 0.1 mm spacing between the lines. Hence, the fabrication of the Ag NSs was a result of two raster scans, occurring at a certain time delay (horizontal raster scans followed by vertical raster scans). After the completion of the first raster scan in ~5 min, the second raster scan started vertically to the earlier one and was completed in another ~5 min. For the raster scanning case, the number of pulses was 10/s and the stage speed was 0.5 mm/s. This resulted in the irradiation with 20 pulses per unit length (1 mm). Initiating with 12 mJ energy (measured just before the axicon lens), three more pulse energies were utilized to perform energy-dependent LAA. In this process, four NSs were fabricated with different incident energies, labelled as AgNS1- 12 mJ, AgNS2- 16 mJ, AgNS3- 20 mJ, and AgNS4- 24 mJ. To achieve a clear representation of the BB imprint, single spot ablation was also performed at the start of the scan for 5 s (estimated number of pulses = 50) corresponding to each of the aforementioned energies. We believed that the cross-raster scan might result in homogenous nanostructure formation on the Ag surface.

Subsequent to the ablation, extensive characterization of the fabricated NSs was performed. The topographical characteristics of these four NSs were systematically assessed with a field emission scanning electron microscope (FESEM)—ZEISS ULTRA 55 (Graz, Austria), acquiring images at different magnifications (sub-micron to sub-200 nm). The single spot-ablation regions, confirming the Bessel profile imprints (central lobe along with the concentric rings) on the metal surfaces, were also carefully characterized. EDX spectra were also recorded to confirm the presence of any oxidation effect. To obtain further nanoscale-topographical features, atomic force microscopy (AFM) was also carried out thoroughly for all NSs with a HITACHI AFM 5000-SPA400 instrument (Tokyo, Japan). In addition, reflectivity measurements were also carried out with a UV-VISIBLE-NIR spectrometer in two incident angles reflection mode (Carry 5000 with UMA attachment, Agilent Technologies, Santa Clara, CA, USA) within the range of 250–1200 nm. Seven analytes were dissolved in methanol/water to perform the SERS studies by simply drop-casting them on the prepared SERS substrates. The analytes used were picric acid (2,4,6-trinitrophenol, C_6_H_3_N_3_O_7_, a potential explosive compound), TH—thiram (C_6_H_12_N_2_S_4_, a pesticide), MB—Methylene blue (C_16_H_18_ClN_3_S, a dye), AN—ammonium nitrate (NH_4_NO_3_), a high energy compound used with explosives; MG—Malachite green (C_23_H_25_ClN_2_), an aquaculture dye compound, NB—Nile Blue (5 µM, C_20_H_20_ClN_3O_) a dye molecule, and also a bio-protein-molecule, HEWL—Hen Egg White Lysozyme. Explosive samples were provided by the HEMRL (High Energy Materials Research Laboratory), Pune, India, whereas the pesticide and dyes were purchased from M/s Sigma Aldrich (St. Louis, MO, USA). The biomolecule (HEWL) was collected from our collaborator at the school of life science, University of Hyderabad. The detailed synthesis process of the HEWL can be found in the supporting information (SI), described in Figure S1 on page S4. All the SERS measurements were performed with an i-Raman plus portable Raman instrument (B&W Tek, Canal Fulton, OH, USA) with optical fiber signal collection. The SERS experiments were performed with 102 mW (30% of full power) excitation power, 5 s of accumulation time, number of spectral averaging 3. The portable Raman consists of a 785 nm excitation laser with ~90 µm spot size. the SERS Mapping studies were carried out with LabRAM HR Evolution, Micro Raman spectrometer (M/s Horiba France SAS, Palaiseau, France) with 50× objective, three excitation wavelengths of 532 nm, 633 nm, and 785 nm. The resolution of the instrument was 3–5 cm^−1^ depending on the spectral region and all the peaks identified in each of the analytical molecules were well resolved, there was no overlap. The gold (Au) coating was performed using a thermal vapor deposition technique. Typically, 10 nm, 20 nm, 30 nm thin layers of gold were deposited on three identical AgNS2 substrates using a thermal evaporation chamber operated at a vacuum pressure of 4 × 10^−6^ mbar pre-deposition and 6 × 10^−5^ mbar during the deposition. The nominal thickness of the film was monitored using the quartz crystal thickness monitor equipped inside the chamber.

## 3. Results

Optical beams involving distinct intensity distribution patterns have always accomplished lively interest among physicists. Among various such kinds, non-diffracting beams have left a momentous mark in this field. The zeroth-order Bessel function of the first kind, elaborating the non-diffracting BB, was extracted originally as the first non-singular solution of scalar Helmholtz wave equation by Durnin in the year 1987 [47,48]. Durnin et al. have approached with an approximation for generating Bessel beams (quasi-Bessel beam), instead of an ideal case (hardly possible to optically achieve) constituted with an infinite number of rings, essentially having required infinite energy. Gori et al. introduced the Bessel–Gauss beam with finite energy [49]. One of the outstanding characteristics of a non-diffracting beam turns out to be the strong resistance of its central maxima towards diffractive spreading along with spatial propagation, unlike all other waves. Comparatively higher depth of focus is offered by the axicon-employed BB, providing a great advantage for laser–matter interaction applications. Similarly, along with a high peak intensity at the central maxima, the BB profile delivers concentric rings having π-phase shifts from one to another. The invariance in the central peak full-width half maxima (FWHM) in case of a zeroth-order BB, compared with increasing nature of the same in case of a Gaussian beam, along the Rayleigh length, has been established well in the reports of Stoyanov et al. [14]. In addition, the self-healing characteristic of the BB also provides a superior advantage in a significant optical application, which has been thoroughly studied by various groups [50]. Coming across an obstruction in its path of propagation, BB can regain its unhindered profile past the object. The development of constructive interference among the multiple coherent plane waves proceeding at an equal angle w.r.t the optical axis can be held accountable for the self-healing beam property [51,52]. Exploiting these key advantages of BB, having a consistent transverse profile a zeroth-order BB was implemented for LAA on a plasmonic silver substrate. In this investigation, we have generated a BB employing an axicon, in order to achieve a far smoother intensity variation rather than the annular slit method (conical lens) [7]. An in-depth theoretical perspective of the BB has been elaborated in the Supplementary Materials file (page S3).

### 3.1. Fabrication of the Ag NSs

**Figure 2:**
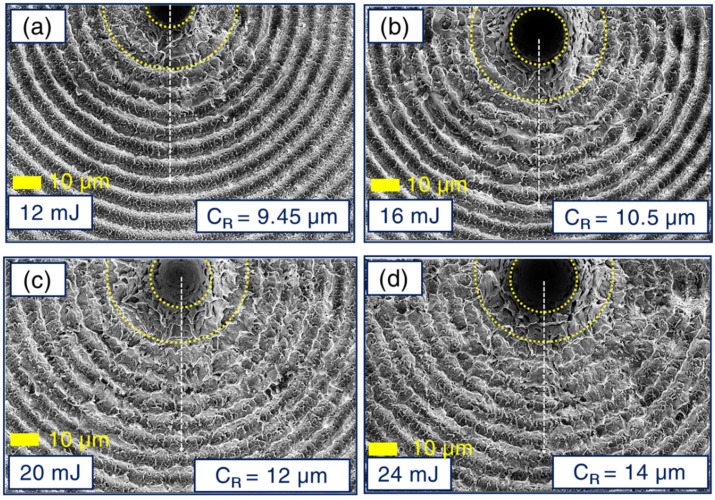
Bessel profile imprints on the Ag substrate in identical experimental and substrate surface conditions with varying pulse energies (**a**) 12 mJ, (**b**) 16 mJ, (**c**) 20 mJ, (**d**) 24 mJ. C_R_ values represent the value of central lobe radii. The first yellow dotted line denotes the central lobe for the estimations of C_R_. The second yellow dotted line exhibits the starting of the circular pattern formations on the Ag surface due to the BB profile. The dotted line depicts the increasing ring radii. We estimated that 50 pulses were incident at this spot.

Distinctive features in the obtained structures prevailed in the case of axicon employed BB ablation in the air over well-explored Gaussian beam induced ablation. Further, the Gaussian beam delivers ~50% of its energy within its FWHM, unlike BB, transferring a major part of its energy through its concentric rings [53]. This makes it conspicuously interesting in the case of laser–matter interaction, leaving a unique imprint of its beam profile on the deposition spot of the material (see Figure 2). To gain a fundamental insight into the intrinsic phenomena occurring in BB cross-raster ablation, leading to the formation of exotic Ag NSs, the surface profiles from the starting irradiation spot (irradiation was up to 5 s, i.e., under the influence of 50 pulses since 10 Hz repetition rate was employed) was meticulously assessed through FESEM images. At the time of performing cross-raster ablation (1.1 cm × 1 cm), different pulse energies were applied (12 mJ—AgNS1, 16 mJ—AgNS2, 20 mJ—AgNS3, 24 mJ—AgNS4) to induce NSs over an area of 4 mm × 3 mm. Increasing order in the radius of central lobes (C_R_) (increasing C_R_ values exhibited in Figure 2), corresponding to increasing pulse energies, was evidently observed. The depth profiles for each central lobe spots were also inspected, depicting an increasing behaviour w.r.t ablation energy (presented in the Supplementary Materials Figure S2). Further insight into the formation of NSs revealed the effect of cross-raster scans on the plasmonic material under interaction. The BB profile is quite evident from its post-ablation signature imprinted on the Ag surface (Figure 2).

**Figure 3:**
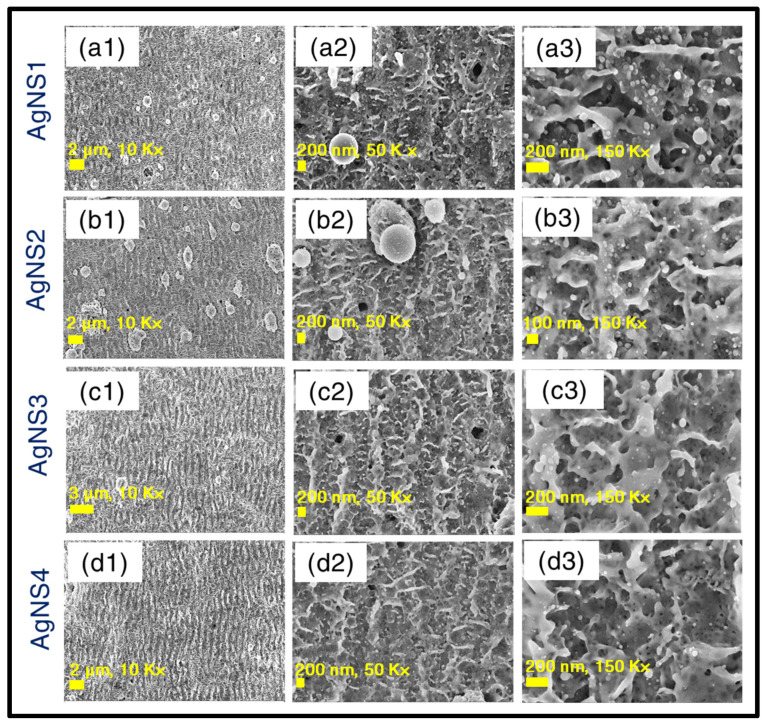
FESEM images for AgNS1 ((**a1**–**a3**)-12 mJ), AgNS2 ((**b1**–**b3**)-16 mJ), AgNS3 ((**c1**–**c3**)-20 mJ), AgNS4 ((**d1**–**d3**)-24 mJ), respectively. For the four sets of images for each NS, in detail, all figures corresponding to labels (1), (2), (3) illustrate FESEM images with different magnifications of 10 K×, 50 K×, 100 K×, respectively.

Hence, in the case of a cross-raster scan, there arises a clear possibility of superposition of two BB profile-induced ablation affected zones. This superposition of basically two BB-affected regions is assumed to happen within a certain time delay, i.e., the time separation between the first raster scan and the second cross-raster scan. To demonstrate the mechanism used an explicit explanation using a schematic representation has been provided in Figure 1. The central lobe diameter was ~10 µm, the complete spot radius turned out to be around ~50 µm (observed from the data presented in Figure 2). The line spacing was ~100 µm and it satisfied the condition of overlapping of BB profile from both sides at the time of the first raster scan (presented in schematic Figure 1d). This overlapping phenomenon was repeated, but in the vertical direction, at the time of the second raster scan, leaving a complete laser-irradiated nanostructure region. This successive laser interaction between the first-raster scan ablated NS with the subsequent ablation from the second raster scan turns out to be a crucial phenomenon and can be attributed as a key reason behind the occurrence of laser-induced fusion of ablated materials in the nanoscale regime and regrowth/re-formation of spherical, aspherical nanoentities (micro, nanobeads shown in Figure S3 and finger-like growths shown in Figure S4, in the Supplementary Materials file) inside the NSs. For each corresponding energy, thorough FESEM images (see Figure 3) have been acquired with different magnifications to obtain a clear picture of the fabricated NSs. Figure 3a1–d1 illustrates the lower magnification pictures, whereas higher magnification figures are presented in Figure 3a2–d2 (50× magnification) and Figure 3a3–d3 (100× magnification). The existence of sub 200 nm nanofinger-like formations was observed in all NSs, which we believe is a significant reason behind the fine SERS response (presented in subsequent sections). The formation of a higher density of re-growths, especially in the case of AgNS2 was confirmed definitively from the 10× FESEM images in Figure 3a1–d1 of all four NSs- AgNS1, AgNS2, AgNS3, and AgNS4. At 16 mJ ablation energy, the presence of a higher number of micro-beads (having 1–2 µm width) can be observed compared to other energy ablated NSs. The formation of these silver beads in higher density all over the AgNS2 is considered as a crucial reason for better plasmonic response. Further, the voids/hierarchal structures in these substrates can also influence the analyte molecule presence during the SERS investigations. We have also performed a qualitative study trying to understand the surface roughness of the NSs and the data are presented in Figure S5a1–d1 of the Supplementary Materials file. To estimate the surface roughness of the various energy-dependent NSs, corresponding FESEM images were fed into Gwyddion software (Czech Metrology Institute, Jihlava, Czech Republic).

No occurrence of oxidation in all structures was confirmed through EDX spectral data, which are presented in Figure S5a2–d2 in the Supplementary material file, page S8. We stored all the samples (post-ablation) in air-tight containers and in desiccators. We removed the samples only when the characterization studies were performed, thereby strongly limiting the oxidation effects. Extensive FESEM images are presented in the supporting information Figures S3 and S4, particularly for the Ag NS2, portraying different nano growth formations in terms of lower, mid-range, and higher magnification images. Subsequently, to obtain an insight into the nanoscale-roughness information, the afore-mentioned NSs were further probed with the atomic force microscopy (AFM) technique. The data are presented in Figure S6 of the Supplementary material file. Subsequently, UV-VIS-NIR reflectivity measurements were also conducted with all the four NSs, along with a pristine Ag substrate for clear comparison. The complete data are elaborated in the Supplementary material file, Figure S7. The obtained results from the present work are in good agreement with the response obtained from Ag nanostructures, fabricated in different methods, as elaborated in previous reports [54,55,56]. Subsequently, the fabricated NSs were used as SERS substrates for the real-time sensing of diverse molecules.

### 3.2. Fabrication of Au-Coated AgNSs

**Figure 4:**
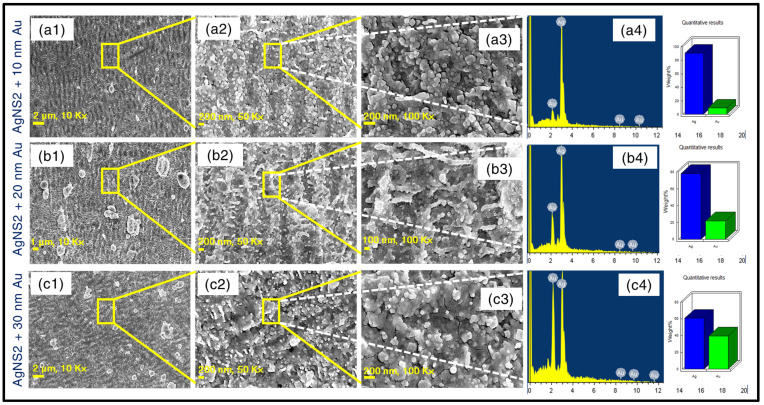
FESEM images along with EDX spectra for three Au coated identical AgNS2 substrates with increasing thickness of Au deposition (**a1**–**a4**) (AgNS2 + 10 nm Au) (**b1**–**b4**) (AgNS2 + 20 nm Au) and (**c1**–**c4**) (AgNS2 + 30 nm Au). For the three set of images corresponding to each Au coated-NS, figures labeled as (1), (2), (3) illustrate the FESEM images with different magnifications of 10 K×, 50 K×, 100 K×, respectively (left to right; e.g., (**a1**–**a3**)). The EDX spectra are displayed in figures (**a4**,**b4,c4**) corresponding to the previous order of coated NSs. The increasing percentage of Au confirmed the increasing thickness of Au deposition.

To accomplish further improvements in the SERS signal, three of the most efficient, identical nanostructures—AgNS2 (ablated with 16 mJ pulse energy) were coated with gold (Au), utilizing different amounts of Au deposition. Gold coating thicknesses of 10 nm, 20 nm, and 30 nm were formed on these three identical AgNS2 SERS substrates. Subsequently, Au-thickness-dependent SERS studies were carried out involving these Au-coated NSs for the trace level sensing of TH molecule. After the development of all three NSs, deposited with growing order of Au thickness, extensive FESEM characterisation studies were conducted. Figure 4a1–a3 depict the 10 nm Au coated Ag NS2 with increasing magnifications of 10×, 50×, 100×, respectively. The minimal amount of growth of Au settled on the AgNS2 is portrayed through these images. Similarly, the nanoscale impressions of 20 nm Au coating on one more identical AgNS2 are portrayed through figures of different magnifications as 10×—Figure 4b1, 50×—Figure 4b2, and 100×—Figure 4b3. The data of the same AgNS2 coated with 30 nm Au are conveyed through Figure 4c1–c3. For the NSs with 20 nm coating, a proper replication of the finger-like structures was observed whereas at 30 nm deposition, the nanoscale roughness was noticed to be overlapped. In the same figure, EDX spectra in Figure 4a4–c4 are also displayed proving a clear picture about the gradual increment in thickness of the Au-coatings. After finalizing the investigations on topographical features of Au-coated AgNSs, they were implemented in sensing studies using diverse analytes.

### 3.3. Sensing Studies Using the SERS

**Figure 5:**
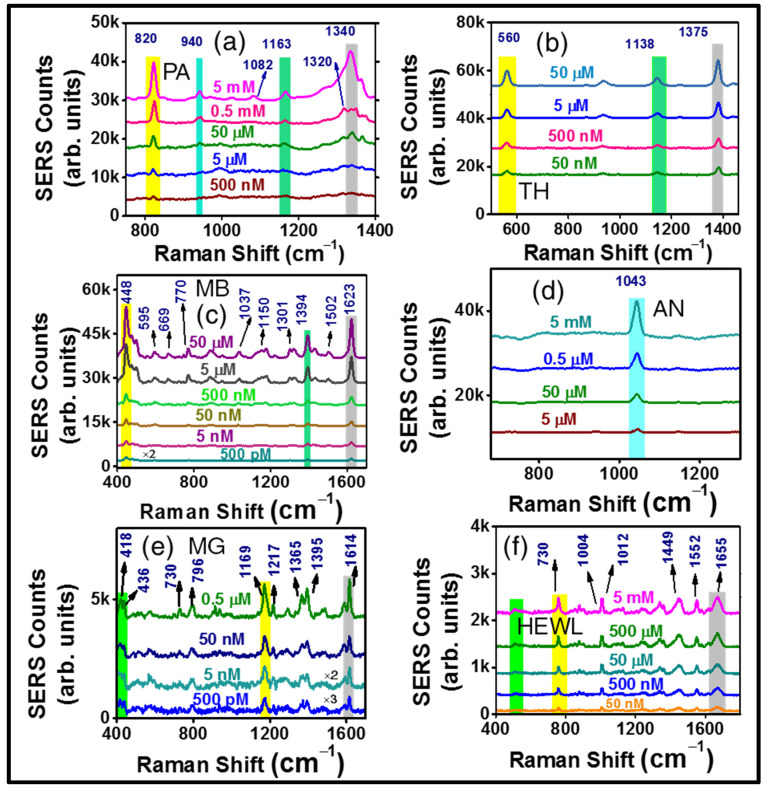
SERS data obtained with six different molecules using portable Raman as well as micro-Raman instrument. Portable Raman spectra were collected with 785 nm, 102 mW (30% of full power) excitation power, 5 s of accumulation time, number of spectral averaging 3, and the laser spot size ~100 µm. Figures demonstrate the concentration dependent Raman spectra for (**a**) PA (500 nM), (**b**) TH (50 nM), (**c**) MB (500 pM), (**d**) AN (5 µM), (**e**) MG (500 pM) molecules, also with their lowest traces. Similarly, figure (**f**) depicts about the concentration varied SERS spectra of HEWL (lysozyme protein biomolecule) collected with 25% of the full power (540 mW) of a 532 nm excitation laser engaged micro-Raman, using an acquisition time of 5 s, accumulation number of 3.

Thus far, various interesting laser-based spectroscopic techniques, including SERS [57,58], have been surveyed to accomplish the purpose of analytes detection, especially explosives. In recent times SERS has emerged as one of the fastest-growing research topics [59,60,61,62,63,64,65,66,67,68,69,70,71,72,73,74], offering fantastic advantages in real-time applications. Surface plasmon resonances in the nano regime, instigating the development of enormous evanescent fields, remain the origin of SERS. In the contemporary research fields of photonics, SERS has made its mark as a fascinating multi-advantageous technique, offering significant applications in hazardous materials sensing. The as-fabricated silver nanostructures were employed as active SERS substrates for trace-level sensing of a number of real-time molecules through the SERS technique. Interestingly, different enhanced responses were observed in the case of each of the four Ag NSs while performing SERS for the trace detection of six different molecules. To reach a decisive conclusion about the plasmonic responses of four NSs, the same were engaged in the detection of three consecutive molecules, PA–Picric acid (2,4,6-trinitrophenol, C_6_H_3_N_3_O_7_, a potential explosive compound), TH–thiram (C_6_H_12_N_2_S_4_, a pesticide) and MB–Methylene blue (C_16_H_18_ClN_3_S, a dye). In the first place, we have engaged a real-time suitable portable Raman spectrometer, with 785 nm excitation, 102 mW (30% of full excitation power), 5000 ms of accumulation time, number of spectra for signal averaging to be 3, and the laser spot size of ~100 µm. The aforementioned parameters were kept unchanged throughout all the SERS measurements in our complete study involving a portable Raman spectrometer. In the rigorous SERS sensing process performed with four NSs, the AgNS2, corresponding to 16 mJ laser ablation pulse energy in air, clearly exhibited the best competence in terms of offering higher electromagnetic (EM) evanescent enhancement, leading to the possibility of lowest trace-level detection. Highly efficient heat-treated drop casting technique has been made use of, to keep a concentrated droplet (typical volume of ~2 µL) of the analyte under investigation. Lowest traces detection of 500 nM, 50 nM, and 500 pM have been achieved through SERS sensing employing the AgNS2, corresponding to PA, TH, and MB, respectively. Figure 5a–c elaborate the extensive concentration varied sensing carried out involving the molecules in the already mentioned order. Even at the lowest concentration of each molecule, corresponding signature Raman peaks are prominently observed. For PA, the Raman signature at 820 cm^−1^ is assigned due to NO_2_ scissoring while at 1338 cm^−1^ it appears because of NO_2_ symmetric stretching. In a similar manner, for TH, S-S stretching occurs near 560 cm^−1^, whereas CN stretching and symmetric deformation CH_3_ are responsible for the appearance of the 1375 cm^−1^ peak. In case of MB, skeletal deformation of C-N-C can be attributed as the reason for 448 cm^−1^ Raman peak and ring stretching of C-C is accountable for 1623 cm^−1^ peak [75,76]. The peak assignments corresponding to different molecular/bond activities is demonstrated for all these molecules and matched well with those presented in the previous reports from our group [75,76,77,78,79,80]. Succeeding the concentration varied study, to produce a survey on the SERS signal homogeneity, an advantageous parameter for real-time detection, relative standard deviation (RSD) of the enhanced Raman signal was estimated by collecting the signal from 30–40 random spots. In correspondence with the other three nanostructures AgNS1, AgNS2, and AgNS3, impressively better signal consistency was experimentally observed while implementing the AgNS2, revealing RSD value of 7–10% in the Raman signal. The 3D reproducibility spectra are displayed in Figure S8 following the order of Figure S8a—PA (5 mM), Figure S8b—TH (5 µM), and Figure S8c—MB (5 nM) in a sequence. Extensive sensing data obtained from AgNS2 are demonstrated entirely in Figure 5. The complete SERS data attained from AgNS1 (12 mJ), AgNS3 (20 mJ), and AgNS4 (24 mJ) can be found in the Supplementary material file Figures S9–S11, respectively. Concentration dependent SERS data revealed the lowest concentration detected with each AgNS1 along with the reproducibility spectra for all detected molecules and RSD data corresponding to the same NS are thoroughly demonstrated in Supplementary material Figure S9.

Similarly, Supplementary material Figure S10 (TH, PA, MB) and Figure S11 (TH, PA) illustrate the complete SERS data acquired with AgNS3 and AgNS4, respectively. For all three molecules (PA, TH, and MB), AgNS2 exhibited lowest trace detected with minimum RSD, achieving enhancement factors (EFs) of 5.7 × 10^4^, 7.3 × 10^5^, and 9.3 × 10^6^, respectively. The enhancement factor calculations have been presented in the Supplementary material file (page S13). Superior localised EM field generation, particularly from the AgNS2, exhibiting SERS response can be justified on the basis of formation of higher density, large-scale-regrowth (micro, nano beads, precisely displayed in Figure S3) overall the ablated nanostructure compared to other NSs (visible in Figure 3a1–d1). Having established the superior performance achieved with particularly AgNS2, the fundamental correlation between the plasmonic response of the various NSs induced by the effect of different ablation energies, and the diversity in their sub ~200 nm structural properties, becomes enlightened. This also led to the further investigation of some additional molecules with unchanged parameters in portable Raman, such as AN—ammonium nitrate (NH_4_NO_3_), a high energy compound used with explosives; MG—Malachite green (C_23_H_25_ClN_2_), an aquaculture dye compound, and also a bio-protein-molecule, HEWL—Hen Egg White Lysozyme. The comprehensive data, including concentration dependent detection of each molecule (for AN (5 mM), MG (50 nM), HEWL (5 mM) sequentially) for the same, are presented in Figure 5. The same AgNS2 was evidently utilized as the SERS substrate for the sequential detection of all of these molecules following an efficient recycling process. A well-organized cleansing technique was followed utilizing multiple solvents, such as methanol and double distilled water, which were primarily used to prepare analyte solutions. The substrates were kept in each solvent for 30 min followed by an ultrasonication process (20 min) in the same solvent. After completion of the cleaning process, any possible presence of the formerly used analyte was verified by acquiring multiple Raman spectra from the same substrate with the portable Raman system. The subsequent measurements were continued only after the confirmation of the absence of any unwanted Raman peaks.

**Figure 6:**
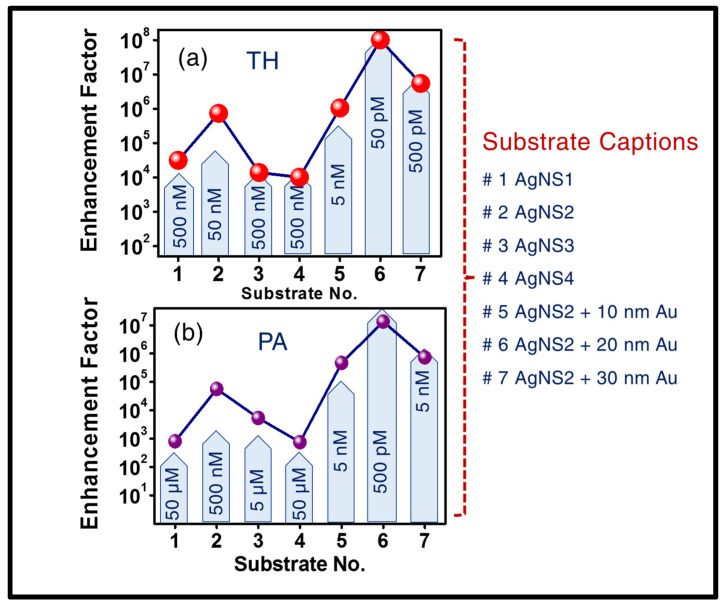
Enhancement factor comparison from the sensing performances of (**a**) TH (**b**) PA. These studies involved all the Ag NSs along with different thickness of Au coated AgNSs. Lowest detection limits (displayed in light blue colour) and enhancement factors (displayed as solid symbols; lines are only a guide to the eye) of each substrate are presented. ‘#’ refers to the sample number.

The trace level of detection was found to be 5 µM for AN (Figure 5d), displaying its signature peak at 1043 cm^−1^ (corresponding to the symmetric stretch mode of NO_3_) and 500 pM for MG (Figure 5e), with its prominent Raman signature at 418 cm^−1^ (out of plane benzene vibration), 1169 cm^−1^ (in-plane C-H bending). The corresponding reproducibility spectra are shown in Figure S8d,e of the Supplementary material file. In this study, the recyclability of the fabricated AgNsS2 is elucidated, making it a potential candidate from a cost-effective application point of view. Furthermore, the versatility of response was reviewed while acquiring surface enhanced Raman signal with the same recycled AgNS2 substrate with HEWL protein biomolecule that exhibited prominent Raman peak at 1655 cm^−1^ up to 50 nM trace, one of lowest reported in the literature of the alike analyte molecule [81,82,83]. Comparing the SERS research data available on HEWL, only this molecule was probed for SERS studies utilizing Horiba micro-Raman spectrometer (LabRAM HR Evolution), with 532 nm excitation, 50× objective, with 25% of total excitation power (~540 mW). The complete data are demonstrated in Figure 5f and S8f. The enhancement factors corresponding to AN, MG, and HEWL molecules were found to be 2.2 × 10^3^, 6.5 × 10^6^, and 1.5 × 10^5,^ respectively. The limits of detection (LoDs) were also estimated, following the standard 3σ/b method, previously reported in our article [75,77], where ‘σ’ is the standard deviation of BB structured substrate and ‘b’ is the slope of the resultant linear plot at lower analyte concentrations. For all the investigated molecules, the linear region at the intensity versus concentration plots corresponding to lower concentrations can be found in the Figure S12, Supplementary material file, page S14. The estimated LoD numbers were impressive for various molecules, such as, PA- 360 nM, TH- 35 nM, MB-300 pM, AN- 3 µM, MG- 210 pM, and HEWL- 27 nM. To present a clear illustration on the superiority of AgNS2 in SERS response in contrast with all four structures, a comparison plot is presented in Figure 6a,b, for thiram and PA, bringing forth a clear distinction in the performance of four NSs, based on enhancement factor (EF) as well as lowest trace detection limit. The summary of detailed study with four NSs, including their corresponding trace concentrations, enhancement factors, and the LoDs are presented in Table 1. While calculating EFs, the Raman spectra were taken on plain, non-structured Ag with a high concentration of analyte (~5 mM). Additional significant parameters, such as the area of the SERS substrate irradiated by 785 nm portable Raman laser, was estimated as ~90 µm, peaks (considered for the EFs estimation) were well resolved (the resolution of the instrument was 3–5 cm^−1^ depending on the spectral region) and signal-to-noise ratio, even when measuring the lowest trace, was found to be 4:1. All the enhancement factors were calculated following our previously reported procedures [77,78,79].

**Figure 7:**
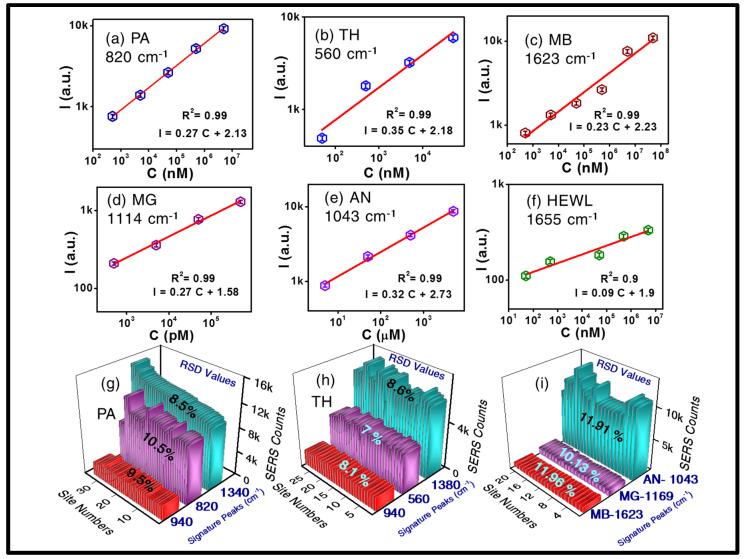
Plots of SERS intensity (I) counts (in arb. units) with respect to analyte concentrations for various molecules (**a**) PA, (**b**) TH, (**c**) MB, (**d**) MG, (**e**) AN, (**f**) HEWL. RSDs were estimated considering 3 major Raman signature peaks of PA (at 820, 940, 1340 cm^−1^) and are presented in (**g**), whereas (**h**) shows the same for TH (at 560, 940, 1380 cm^−1^). Similarly, RSDs for major peaks of MB (1623 cm^−1^), MG (1169 cm^−1^), AN (1043 cm^−1^) are demonstrated in the panel (**i**). Open-hexagons with error bars, in figure (**a**–**f**), depict the intensity counts corresponding to the detected analyte concentration and red line shows the linear-fit.

In due course of an extensive data analysis, the intensity variation with analyte concentration was meticulously assessed by considering the enhanced Raman intensity counts for a particular signature peak corresponding to each molecule and completing the plot with concentration values versus Raman pean intensity. These are the calibration curves and can be used for finding out unknown concentration of the analyte molecule if the Raman peak intensity is measured. These curves were used for estimating the LODs. Figure 7 illustrates the variation of the SERS intensity versus concentration for all the molecules (a) PA, (b) TH, (c) MB (d).

The corresponding 3-D bar graphs exhibit the RSDs of the enhanced intensity counts for all the three signature peaks of PA (Figure 7g). Similarly, the three Raman peaks of TH are presented in Figure 7h and finally Figure 7i summarizes the same about the prominent Raman peaks of MB, MG, and AN, respectively. The error in these data is estimated to be <5% based on multiple measurements. Table 1 summarizes the estimated EFs for various analyte molecules investigated in the present study.

### 3.4. SERS Mapping Studies

**Figure 8:**
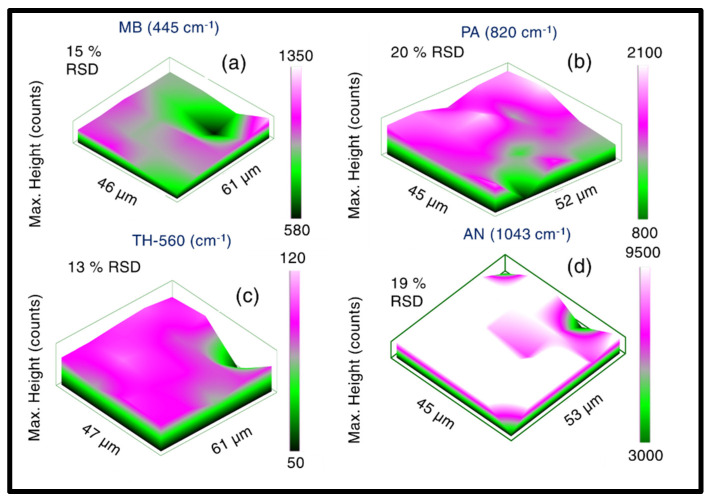
SERS mapping data of AgNS2 obtained with a micro-Raman spectrometer engaging 25% (of full power) of 532 nm laser, a 50× objective, with the following experimental parameters: acquisition time 5 s, accumulation number 3 and a mapping step size of 10 µm. A 3D representation of the Raman-mapped area of ~(45 µm × 50 µm), probing various molecules as (**a**) MB, (**b**) PA, (**c**) TH, (**d**) AN is presented.

After performing extensive sensing investigations using a portable Raman spectrometer, the most efficient substrate AgNS2 was deliberately engaged in Raman mapping exploration studies. In this regard, a micro-Raman spectrometer (LabRAM HR Evolution) was used in the study to access into much smaller resolution of plasmonic hotspots, unveiling the plasmonic character at such sub-µm scale. The difference in signal collection was the fundamental difference between the portable Raman spectroscopy (where ~100 µm laser spot size is employed) and in micro-Raman spectroscopy wherein (~2 µm laser spot size is typically utilized). In the signal mapping acquisition process, 25% laser excitation power of 532 nm laser (~540 mW) was employed with a 50× objective and acquisition parameters were set as follows—acquisition time of 5 s, accumulation number 3, and a mapping step size of 10 µm to avoid any photodegraded portion due to intense excitation. Overall comprehensive signal enhancing responses were retrieved from a significantly large sample areas (40 µm × 50 µm), involving identical analyte molecules utilized in previous studies. The motivation of this study was to investigate the plasmonic response emerging from random and smaller areas of interest and, therefore, the concentrations of the molecules was not constant for all the analyte studies. The complete Raman mapping data are illustrated in Figure 8. Previously detected molecules, such as MB −50 µM in Figure 8a, PA-5 mM in Figure 8b, TH-5 µM in Figure 8c, and AN-5 mM in Figure 8d (presented sequentially) were Raman mapped with 532 nm excitation utilizing the above-stated experimental parameters. For a single signature peak the RSDs of intensity counts were estimated. The accumulation of the Raman signal from very small sections is believed to be major reason for higher RSD compared to portable Raman sensing data. In the portable spectrometer case, the Raman signals were collected from large areas and, therefore, the RSD values are found to be lower. We firmly believe that portable Raman spectrometers are the way forward to perform practical detection in the field. Additionally, the excitation energy dependent response of the plasmonic NS was thoroughly studied and its data are depicted in Figure S13, Supplementary material file. Moreover, the AgNS2 substrate was again employed in the wavelength dependent SERS study, demonstrated in details in Supplementary material file, Figure S14. A gradual increase in the Raman intensity as the excitation wavelength (λ) becomes shorter can be attributed to the λ^−4^ dependence of the Raman scattering signal [84]. However, further extensive study needs to be performed to take in account various other inherent phenomena such as resonance effect, which will be our future goal.

### 3.5. Aging Studies of the SERS Substrates

**Figure 9:**
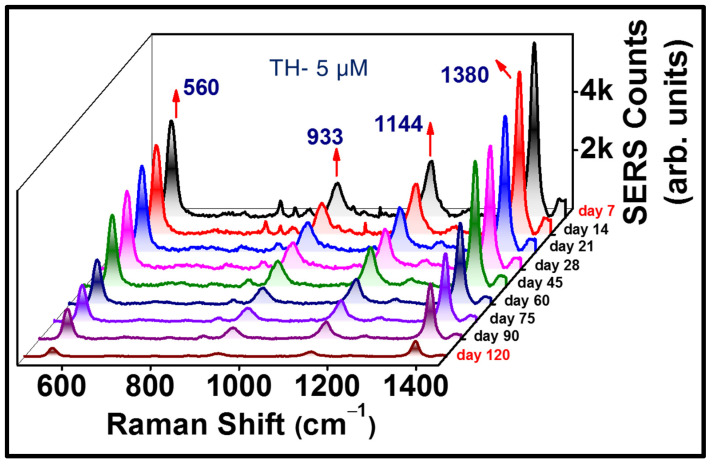
Stability data of AgNS2 while sensing TH (5 µM), illustrating the significant 560 cm^−1^ peak along with others. The SERS data were collected up to 120 days.

To understand the efficacy of our substrates in real-time application point of view, one of the BB fabricated substrates (AgNS2) was engaged in assessment of consistency in its plasmonic character over a long period of time. We pursued to acquire multiple SERS spectra of thiram (at 5 µM concentration) with a portable Raman spectrometer (keeping all the experimental parameters unchanged) over a period of 4 months, with an interval of one week each time. The substrate was cleansed prior to drop casting the exact same amount (~2 µL, 5 µM) of analyte each time, followed by the signal accumulation. Figure 9 explicates the gradual decay in the Raman signal of TH (560 cm^−1^) over a time-span of 4 months. A deterioration in the nanoscale-hotspots of the plasmonic Ag NS, due to multiple cleanings of the substrate, can possibly be held accountable for the reduction in the SERS counts. We clearly observed that 50% of the SERS signal was intact even after 60 days. Furthermore, the generation of prominent signature SERS peaks (with at least 300 counts) even after >120 days of implementation of sensing process clearly signifies the cost-effective nature and real-time utility of our substrates in the merit of practical feasibility.

**Figure 10:**
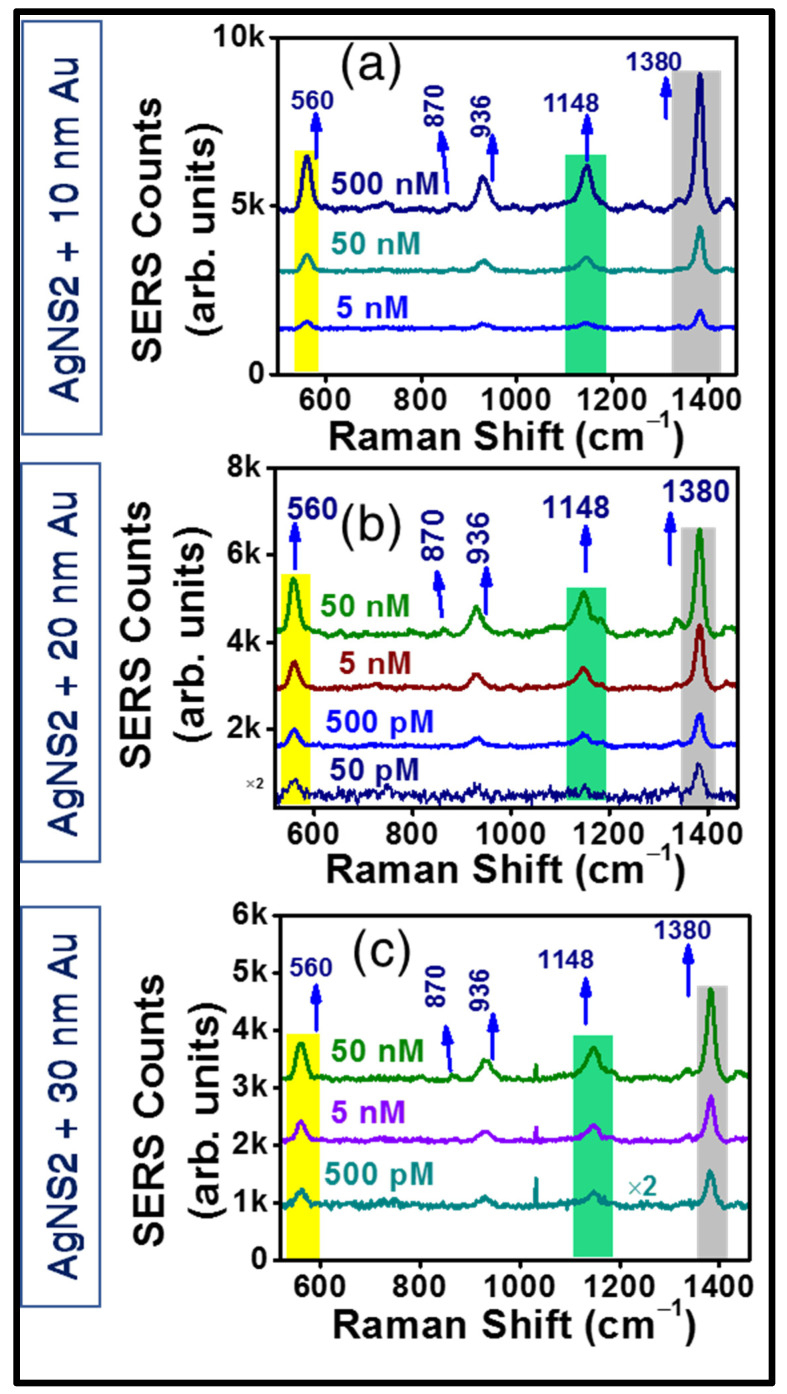
Concentration dependent SERS spectra of Au coated AgNS2 substrates in the detection of TH molecule. The lowest trace limits have been exhibited to be 5 nM from 10 nm coating (**a**), 50 pM from 20 nm coating (**b**), and 500 pM from 30 nm Au deposition (**c**). The best trace detection efficiency was achieved with 20 nm Au coated AgNS2.

Additionally, to reach a decisive conclusion about the plasmonic responses of the three Au-coated substrates (10 nm, 20 nm, and 30 nm coated AgNSs), two of the previous molecules were again detected utilizing the aforementioned coated NSs. Significantly, superior SERS performance is noted for all the coated AgNSs investigated, not only in terms of higher Raman peaks intensity, but also in the achievement of far better homogeneity of signal, and most importantly, detection of further lower traces. In Figure 10, we present the SERS data of TH where superior response was found with the 20 nm Au-coated AgNS, demonstrating the lowest detection limit of 50 pM (see Figure 10b). However, the 10 nm and 30 nm Au-coated NSs were able to detect only up to 5 nM, 500 pM, respectively (Figure 10a,c).

**Figure 11:**
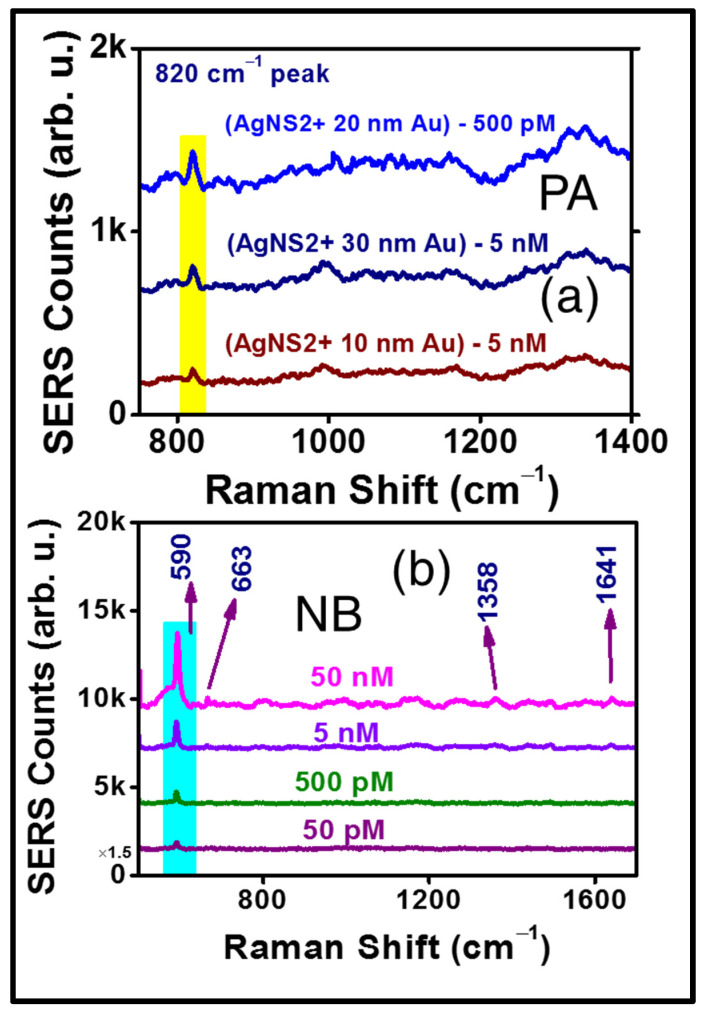
(**a**) SERS spectra of PA molecule, confirming the lowest trace detection was 500 pM with (AgNS2 + 20 nm Au) substrate (**b**) concentration dependent SERS spectra of NB, with 50 pM lowest trace being detected.

The reproducibility of the SERS signal was verified (data are presented in Supplementary material file, Figure S15a–c for 10 nm, 20 nm, and 30 nm Au coated AgNS2, respectively) for each NS, revealed an impressive improvement compared to the uncoated AgNS2. The Au coating served two purposes: (a) it prevented oxidation of the Ag NSs over a long period of time and (b) additional Raman enhancements were achieved from the Au coatings (which themselves are nanostructures now). We believe there has been a cooperative enhancement effect due to this Au coating and we have proposed a mechanism explaining this in the subsequent section. The same three substrates were also utilized for the detection of PA. The complete SERS data of the 20 nm Au-coated AgNS2 detecting PA and NB are presented in Figure 11a,b, respectively. These results decisively concluded the relatively superior performance of the 20 nm Au-coated AgNS, being successful in detecting 500 pM of PA while the other two substrates (10 nm, 30 nm) could detect traces up to 5 nM only. We were successful in detecting another molecule NB up to 50 pM. The concentration dependent SERS spectra of NB are demonstrated in Figure 11b.

Figure S16 (Supplementary material file) illustrates data of the SERS intensity variation with respect to concentration (Figure S16a and also 3D bar graph representations of the RSDs (Figure S16c)), corresponding to the three Au-coated substrates. The reproducibility spectra of NB (see Figure S16b) along with RSD estimations for TH, NB molecules is provided in Figure S16c of the Supplementary material file. An excellent consistency of the Raman signal has been observed from the 20 nm Au coated substrates, offering the best RSD (~4%), whereas 10 nm, 30 nm Au coated AgNS2 substrates also were able to accomplish reasonably RSDs (6.5%, 7.2%, respectively) in the detection of TH. The intensity versus concentration dependent SERS signal of NB is presented in Figure S16d.

The LoD calculations revealed the detection limits for TH, PA corresponding to each Au-coated NSs. A lowest LoD of ~30 pM (TH) has been achieved with the 20 nm Au coated AgNS2 (intensity-concentration graphs for all three coated NS are shown in Figure S17, Supplementary material file) and the corresponding EF was found to be 1 × 10^8^, highest among all the fabricated NSs. The summary of all the EF, LoDs obtained in the work are presented in Table 1. In the detailed model calculation procedure (provided in the Supplementary material file, page S19, S20), followed to estimate the EFs and LOD, Figure S18 illustrates the Raman spectra of thiram (5 mM, in purple), taken on a bare silver substrate. The SERS signal corresponding to lowest possible detected trace of thiram (50 pM, in red) on (AgNS2 + 20 nm Au coating) is also presented. We have also performed a brief comparison of the recently reported SERS substrates with the present work and the data are presented in the Table S1 of the Supplementary material file.

### 3.6. Cooperative Enhancement in the SERS Signal

A sharp EM field generated within the vicinity of plasmonic NSs plays a crucial role to trigger the huge enhancement in scattered Raman signal. Several studies regarding these intrinsic hotspots [85,86,87,88] of the plasmonic nanoentities have emerged out as hot topic of research interest. The interesting phenomena of additional enhancement in SERS signal can be justified by exploring the two-body configuration constituting the AgNS2 interfaced with sub-30 nm gold coatings. We believe that the combination of these two plasmonic systems gave rise to the additional engendering of localised surface plasmon (LSP), leading to augmentation in evanescent electric field offered by the conjugated system. Yang et al. have recently reported a sandwich structure citing significant enhancement in analyte Raman signal. They have demonstrated ‘Au film–molecules–AgNPs’ combined system approach, that turned out to be effective in offering boosted SERS signal for R6G molecule and also similar to a two-body configuration [87]. Liu et al. also reported significant increase in the Raman intensity from a hybrid system consisting of Ag NPs with multilayer Au/Al_2_O_3_ film [88]. In this spectrum of work, we believe that the instigation of the huge field-enhancement from the two-body integrated system (nm-scale Au coating + nm Ag NS) can be explained on the basis of LSP coupling effect unified from both plasmonic systems. This cohesive evanescent field response can be interpreted from the phenomena fundamentally correlated to the penetration depth of any incident electromagnetic (EM) waves [89,90]. Beer–Lambert’s law governs the exponential fall in the intensity of incident EM wave (light) with respect to its penetration depth.

Here,

(1) $$I\left(z\right)=I_{0}e^{-z\alpha \left(\omega \right)}$$

where I(z), $I_{0}$ stands for the penetrated intensity at depth *z* and initial intensity. Optical penetration depth can be well-defined as the propagation distance of an EM wave in a material at which its initial intensity decays to the value of its 1/e. Inverse of attenuation factor is the conventional expression for penetration depth [$\delta_{p}\left(\omega \right)$],

(2) $$\delta_{p}\left(\omega \right)=\alpha^{-1}\left(\omega \right).$$

**Figure 12:**
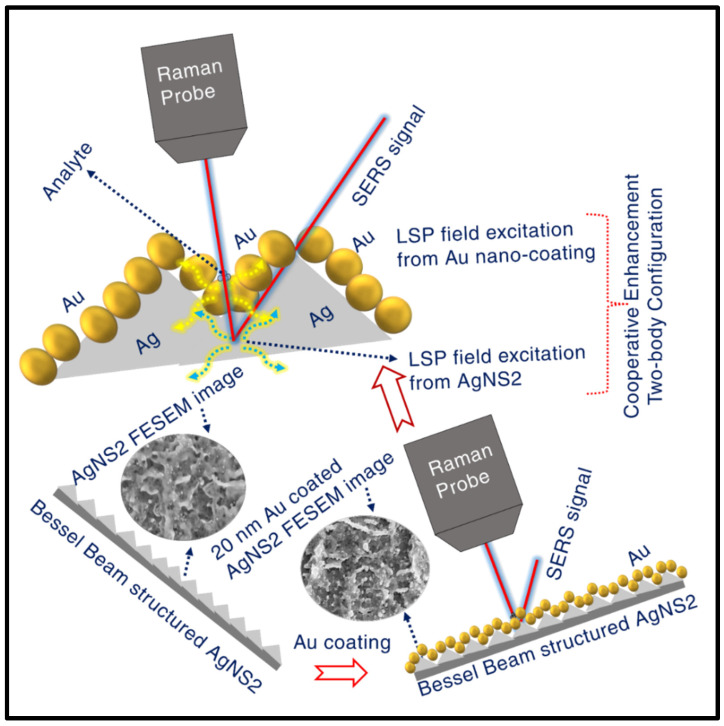
Schematic of Au coated AgNS2 explaining the mechanisms giving rise to cooperative enhancement from two-body system (AgNS2 + 20 nm Au). An evanescent electric field excitation is demonstrated in two-body system due to an appropriate match in penetration-depth condition.

**Figure 13:**
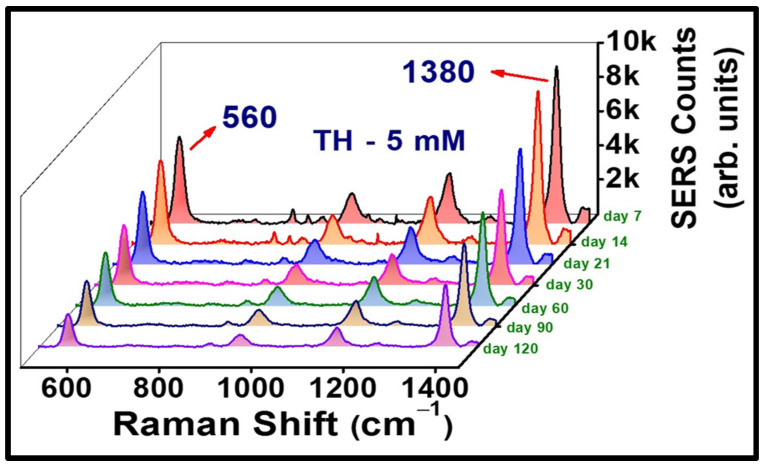
Stability data of the 20 nm Au-coated AgNS2 in sensing TH (5 µM) molecule, illustrating a superior performance compared to bare AgNS2.

Rodrigo et al. have exhibited their work on spectral dependence for different metals of regarding skin depth for an EM plane wave invading at normal incidence on the metal surface [90]. Their observations clearly confirmed the wavelength dependent variation in skin depth of various metals, particularly depicting the optical skin depth of Au, was ~27 nm [90]. This skin depth assessment is believed to be crucial for the occurrence of two-body configuration enhancement. The 785 nm incident excitation is believed to stimulate localised surface plasmonic excitation in both AgNS as well as Au-nano-coating, creating local evanescent–electric field coupling from the combined interactive plasmonic system. Having the skin depth of the normally incident light ~27 nm, higher than the 10 nm, 20 nm Au coating on AgNS, we do believe that the excitation pump is efficient to interfere in the Ag NS region through the Au coated section, inducing LSP in both segments. As a resultant of the field-excitement in the combined system, the superior enhancement is accomplished through the coupled field contribution from the integrated system (schematically represented in Figure 12). The reason behind the aforesaid optimum coupling (leading to maximum SERS response) attained with 20 nm Au coating can be established based on enriched presence of Au layer on the top of AgNS, developing exotic finger-like growths (see Figure 4b2, b3), ideal for the appearance of huge SERS signals. In case of 10 nm coating, the nano-growths were observed (can be observed Figure 4c2, c3) to be not so sharp enough to produce increased evanescent-intensity impact, leading to not-so-enhanced SERS signal. However, the lower-scale enhancement due to the 30 nm Au coated NS could be substantiated on behalf of the thickness scale that is found to be reasonably excessive to complement the skin-depth condition vis-à-vis 785 nm excitation. Figure 13 shows the stability data of the Au-coated AgNS2 in sensing TH (5 µM) molecule, demonstrating a superior performance compared to bare AgNS2. The peaks magnitude was down only to 30% of the original counts even after 120 days of testing. This clearly demonstrates the utility of these substrates in the long run. We anticipate further improvements in these numbers by packing the substrates in vacuum-sealed covers immediately after ablation. Further studies supporting this argument are in progress. Additionally, a comparison between the BB ablation and Gaussian beam ablation is warranted to identify the strengths of the BB ablation and will be a focus of our future studies.

## 4. Conclusions

Laser ablation of a silver substrate in ambient air and under the influence of picosecond Bessel beam profile was carried out for the first time, to the best of our knowledge. Earlier experiments emphasized the advantages of BB ablation over conventionally employed Gaussian beam [91]. The BB profile induced single spot ablation imprint on Ag as well as the variation of its central lobe region have been thoroughly explored. The unique ablation mechanism of silver, involving the superposition of two ps BB beam profile triggered ablation zone has been thoroughly investigated. These structures were meticulously characterised using the FESEM, AFM, and UV-visible-IR techniques. Furthermore, the fabricated NSs exhibited an excellent performance in the sensing activities behaving as versatile, recyclable, stable, reproducible SERS substrates in tracing various hazardous molecules. Some of the highlights are summarized as follows:

- A qualitative correlation was found between the nano-structural surface roughness and the disparity in plasmonic responses of the different Ag NSs, formed engaging systematically increasing laser pulse energies. Additional detailed studies over large surface areas of these substrates will enable us to quantify the surface roughness and the correlation with SERS performance. Subsequently, excitation energy, wavelength and time scale dependent, wide-ranging Raman studies, were performed engaging the most efficient AgNS2.
- Typical enhancement factors of 5.7 × 10^4^, 7.3 × 10^5^, and 9.3 × 10^6^ were achieved for the three hazardous molecules of PA, TH, and MB, respectively, using the AgNS2.
- The LOD’s for TH, PA, MB, AN, MG, and HEWL were estimated to be 35 nM, 360 nM, 300 pM, 3 µM, 210 pM, and 27 nM, respectively.
- Furthermore, the Raman mapping was performed on that substrate providing an insight to the uniformity of the Raman signal sub-micron scale SERS response. We obtained reasonably good RSD values.
- An attractive improvement in the sensing efficiency was accomplished through optimised Au coating on the AgNS2, originating from the two-body-cooperative (AgNS2 + Au) LSP coupled enhancement SERS signal.
- EF’s of 10^8^ (and a corresponding LOD of 30 pM) was achieved for AgNS2 + 20 nm Au coating. Without coating the corresponding numbers were 7.3 × 10^5^ (35 nM).
- The stability of the substrates was found to be improved post Au coating and we observed only ~70% reduction only in the SERS intensities over a period of 120 days in the coated substrates.
- The batch-to-batch variations in the response from the substrates were carefully investigated and the standard deviation in the SERS counts for a particular analyte (fixed concentration) of our interest were collected from three Ag substrates (produced with same ablation conditions). We found it to be within the experimental error of 100 counts, demonstrating a relative standard deviation (RSD) of <6%.
- For the molecules of thiram, PA, MB we have obtained the highest EFs and smallest LODs among a horde of substrates [40,41,42,43,44,45,46,47,48,49,68,69,79,80,81,82,83] investigated by our group in the last 10 years.

## Figures and Tables

**Table 1:** Summary of the estimated AEFs for various analyte molecules involving all the substrates investigated in the present study. Here, AgNS1, AgNS2, AgNS3, AgNS4.

SERS Substrate	Analyte Molecules	Peak Position (cm^−1^)	Lowest Detected Concentration	Analytical Enhancement Factor (AEF)	Limit of Detection (LoD)
**AgNS2**	TH	560	50 nM	7.3 × 10^5^	35 nM
	PA	820	500 nM	5.7 × 10^4^	360 nM
	MB	1623	500 pM	9.3 × 10^6^	300 pM
	AN	1043	5 µM	2.2 × 10^3^	3 µM
	MG	1169	500 pM	6.5 × 10^6^	210 pM
	HEWL	1655	50 nM	1.5 × 10^5^	27 nM
**AgNS2 + 10 nm Au coating**	TH	560	5 nM	1 × 10^6^	2 nM
	PA	820	5 nM	4.7 × 10^5^	-
**AgNS2 + 20 nm Au coating**	TH	560	50 pM	1 × 10^8^	30 pM
	PA	820	500 pM	1.3 × 10^7^	-
	NB	590	50 pM	8.7 × 10^7^	33 pM
**AgNS2 + 30 nm Au coating**	TH	560	500 pM	5.5 × 10^6^	200 pM
	PA	820	5 nM	7.3 × 10^5^	-
**AgNS1**	TH	560	500 nM	3.1 × 10^4^	-
	PA	820	50 µM	8 × 10^2^	
**AgNS3**	TH	560	500 nM	1.4 × 10^4^	-
	PA	820	5 µM	5.3 × 10^3^	
	MB	1623	5 nM	9.6 × 10^5^	
**AgNS4**	TH	560	500 nM	1 × 10^4^	-
	PA	820	50 µM	7.5 × 10^2^	

## Data Availability

Not applicable.

## Supplementary Materials

The following supporting information can be downloaded at: https://www.mdpi.com/article/10.3390/ma15124155/s1, Description: Theoretical aspects of the physics involving Bessel beam; Figure S1: Lysozyme solution preparation and characterization; Figure S2: Depth profiles of the central lobe regions of Bessel beam induced single spot ablation zones; Figure S3: Lower and mid-range magnification FESEM images from a number of spots in Ag NS2; Figure S4: Higher magnification FESEM images from a number of spots in Ag NS2 nanostructure; Figure S5: Surface roughness analysis and EDX measurements; Figure S6: AFM studies; Figure S7: Reflectivity studies concerned with Ag NSs; Figure S8: SERS reproducibility spectra; Figure S9: SERS data collected with AgNS1 (12 mJ); Figure S10: SERS data collected with AgNS3 (20 mJ); Figure S11: SERS data collected with AgNS4 (24 mJ); Description: Enhancement factor (EF) Calculation; Figure S12: SERS intensity variation plot for uncoated AgNS with analyte concentration for LoD estimations; Figure S13: Excitation intensity dependent plasmonic response of the Ag NS; Figure S14: Wavelength dependent SERS studies; Figure S15: Reproducibility spectra for thiram with three Au-coated AgNS2; Figure S16: Logarithmic intensity-concentration plot, RSD plot for three different thickness coated AgNSs; Figure S17: SERS intensity- concentration plots for coated NSs for LoD estimations; Figure S18: Enhancement Factor calculations; Table S1: Sensing comparison of the recently reported SERS substrates with the present work.
