# Supplementary material for: Picosecond Bessel Beam Fabricated Pure, Gold-Coated Silver Nanostructures for Trace-Level Sensing of Multiple Explosives and Hazardous Molecules
Source: Materials (Basel). 2022 Jun 11;15(12):4155. doi: 10.3390/ma15124155 (PMC9228845; doi:10.3390/ma15124155)
Supplement: Supplementary file 1 [file materials-15-04155-s001.zip › materials-1704384-supplementary.pdf]

materials

MDPI

Supporting Information for

Picosecond Bessel Beam Fabricated Pure, Gold-Coated Silver Nanostructures for Trace-Level Sensing of Multiple Explosives and Hazardous Molecules

Dipanjan Banerjee [1], Mangababu Akkanaboina [2], Subhasree Ghosh [3] and Venugopal Rao Soma [1,*]

[1] Advanced Centre of Research in High Energy Materials (ACRHEM), University of Hyderabad, Hyderabad 500046, Telangana, India; 19acpp02@uohyd.ac.in
[2] School of Physics, University of Hyderabad, Hyderabad 50006, Telangana, India; 17phph19@uohyd.ac.in
[3] Department of Biotechnology and Bioinformatics, School of Life Sciences, University of Hyderabad, Hyderabad 500046, Telangana, India; 17ltph17@uohyd.ac.in
* Correspondence: soma_venu@uohyd.ac.in or somavenu@gmail.com

Table of Contents

Serial No.	Caption	Description of content	Page No
**1**	**Description**	Theoretical aspects of the physics involving Bessel beam	3
**2**	**Figure S1**	Lysozyme solution preparation and characterization	4
**3**	**Figure S2**	Depth profiles of the central lobe regions of Bessel beam induced single spot ablation zones	5
**4**	**Figure S3**	Lower and mid-range magnification FESEM images from a number of spots in Ag NS2	6
**5**	**Figure S4**	Higher magnification FESEM images from a number of spots in Ag NS2 nanostructure	7
**6**	**Figure S5**	Surface roughness analysis and EDX measurements	8
**7**	**Figure S6**	AFM studies	8–9
**8**	**Figure S7**	Reflectivity studies of the Ag NSs	9–10
**9**	**Figure S8**	SERS reproducibility spectra	11
**10**	**Figure S9**	SERS data collected with AgNS1 (12 mJ)	12
**11**	**Figure S10**	SERS data collected with AgNS3 (20 mJ)	12
**12**	**Figure S11**	SERS data collected with AgNS4 (24 mJ)	13
**13**	**Description**	Enhancement factor (EF) calculation details	13
**14**	**Figure S12**	SERS intensity variation plot for uncoated AgNS with analyte concentration for LoD estimation	14
**15**	**Figure S13**	Excitation intensity dependent plasmonic response of the Ag NS	15
**16**	**Figure S14**	Wavelength dependent SERS studies	16
**17**	**Figure S15**	Reproducibility spectra for Thiram with three Au-coated AgNS2	17
**18**	**Figure S16**	Logarithmic intensity-concentration plot, RSD plot for three different thickness coated AgNSs	18
**19**	**Figure S17**	SERS intensity- concentration plots for coated NSs for LoD estimations	19
**20**	**Figure S18**	Enhancement Factor calculations	19–20
**21**	**Table S1**	Sensing comparison of the recently reported SERS substrates with the present work	S22

Theoretical aspects of the physics involving Bessel beam:

Due to the incidence of a Gaussian laser beam on the conical lens, the beam geometrically interacts with itself [see figure 1(b)] after getting congregated to the axis of symmetry, resulting in a distinctive Bessel beam shape. The ideal Bessel beam can be described by,

$$\boldsymbol{E(r,\varphi,z) = A_0 \exp(ik_z z) J_n(k_r r) \exp(\pm in\varphi)} \quad \text{(S1)}$$

$\boldsymbol{J_n}$ is an nth order Bessel function, $\boldsymbol{k_z}$, $\boldsymbol{k_r}$ being the longitudinal and transverse wavevectors and $\boldsymbol{k = \sqrt{k_z^2 + k_z^2} = 2\pi/\lambda}$, λ denotes the EM wave length constituting the Bessel beam and r, $\boldsymbol{\varphi}$, z, are the radial, azimuthal and longitudinal components, respectively.

The depth of focus (DOF) of the employed axicon, being function of incident beam radius (r), is defined mathematically as,

$$\boldsymbol{DOF = \frac{r\sqrt{1-n^2 \sin\alpha}}{\sin\alpha \cos\alpha (n\cos\alpha - \sqrt{1-n^2 \sin\alpha})}} \quad \text{(S2)}$$

Where, n (=1.458), α (=10^0) appears to be axicon refractive index, alpha angle of the conical optic respectively. In the view of a non-absorbing axicon, illuminated by a Gaussian beam, the expression for optical intensity distribution past the axicon can be found as

$$\boldsymbol{I(\rho,w) = \frac{I_0 \pi\beta w}{2}[\{(F_1(\rho/w) + F_2(\rho/w))J_0(\rho\beta))\}^2 + \{(F_1(\rho/w) + F_2(\rho/w))J_1(\rho\beta))\}^2]} \quad \text{(S3)}$$

where J_0, J_1 are the first kind of Bessel function, zeroth and first order respectively and the F_1, F_2 are mathematical functions as, $\boldsymbol{F_1(\rho,w) = (z_0 + \rho/w)^{1/2} \exp[-(z_0 + \rho/w)^2]}$; $\boldsymbol{F_2(\rho,w) = (z_0 - \rho/w)^{1/2} \exp[-(z_0 - \rho/w)^2] H(z_0 - \rho/w)}$; $\boldsymbol{z_0 = (n-1)\alpha z/w}$, H being the Heaviside step function and propagation length = $\boldsymbol{z_{max} = w/(n-1)\alpha}$. In case of pulsed lasers, separating the time dependent term, the fluence can be described as [1],

$$\boldsymbol{F(\rho,w) = \frac{Q\beta}{w}[\{(F_1(\rho/w) + F_2(\rho/w))J_0(\rho\beta))\}^2 + \{(F_1(\rho/w) + F_2(\rho/w))J_1(\rho\beta))\}^2]} \quad \text{(S4)}$$

where in Q (in Joules), w (in m), β (m^{-1}) is signified as pulse energy, w is the half-width of the Gaussian beam waist. Evidently, the maximum fluence, observed at the central lobe portion of the Bessel beam, can be expressed as,

$$\boldsymbol{F = \frac{2Q\beta}{w\sqrt{e}}}$$

(S5)

$$\boldsymbol{\beta = \frac{2\pi(n-1)\alpha}{\lambda}}$$

(S6)

'n' is the refractive index of the axicon lens materials, α is the apex angle of the same.

Lysozyme solution preparation and characterization:

HEWL powder was dissolved [20 mg/ml] in double distilled water and the pH was adjusted to 7. Protein stock concentration was determined by UV absorbance measurements in Cary100 absorbance spectrophotometer equipped with a temperature-controller. The 100 times diluted protein sample was used to record the spectrum with the rate of 60 nm/min using 10 mm path length cuvette and the protein concentration was measured using the extinction coefficient of 38940 $M^{-1}cm^{-1}$ at 281 nm and the Beer-Lambert law. From this stock solution 1 mM, 500 μM, 50 μM, 500 nM and 50 nM protein dilutions were prepared for the further study. The purity of HEWL was confirmed by the presence of a single band by sodium dodecyl sulfate poyacrylamide gel electrophoresis (SDS PAGE). For far-UV circular dichroism (CD) spectra 15 μM HEWL in double distilled water (pH 7) was used and the experiment was carried out in Jasco-J1500 spectropolarimeter connected to a water-bath. The spectrum was collected with the scan rate of 20 nm/min using 2 mm path length cuvette for far-UV measurements from 250 to 195 nm with 2 accumulations. For recording both UV absorbance and far-UV CD spectra, double distilled water (pH 7) was used as a blank correction.

(**a**) (**b**)

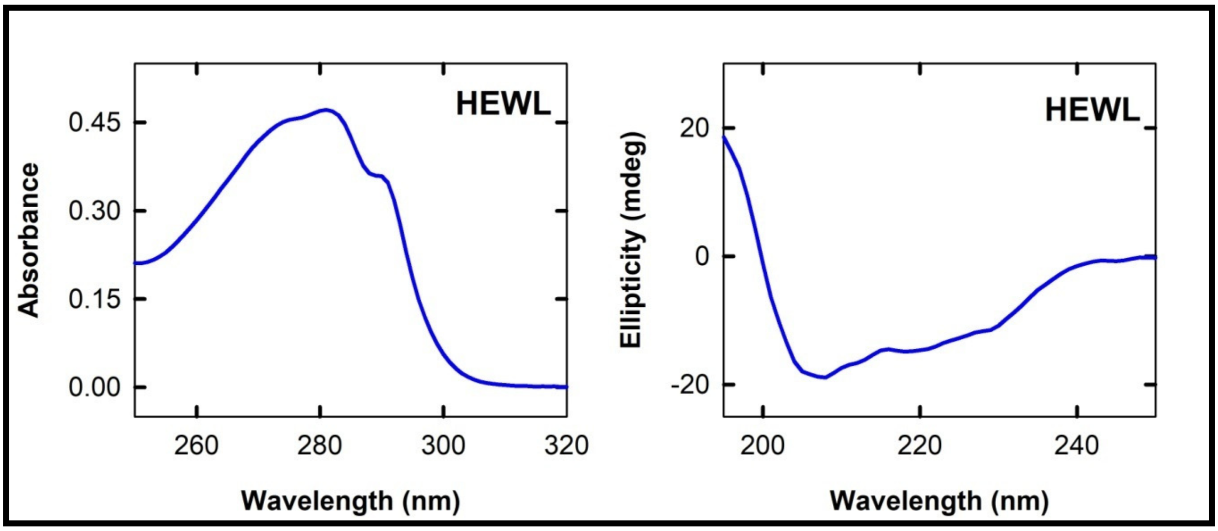

Figure S1 (a) UV absorbance spectrum of HEWL measured at room temperature and (b) Far-UV CD spectra of HEWL measured at room temperature.

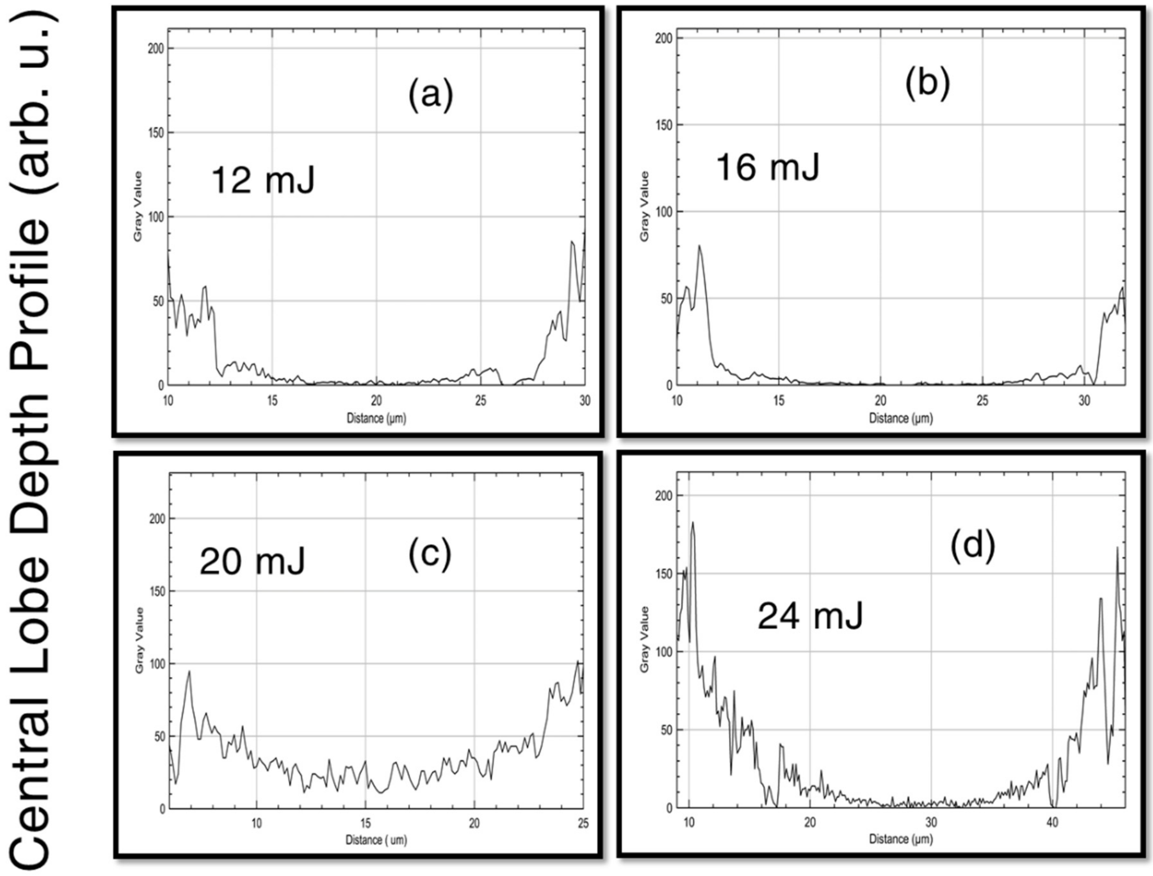

Figure S2 Depth profiles of the central lobe regions of Bessel beam induced single spot ablation zones (at the start of the scan) corresponding to (**a**) 12 mJ (**b**) 16 mJ (**c**) 20 mJ (**d**) 24 mJ pulse energies. Depth profiles have been estimated by taking a line profile from the FESEM images (figure 2) through Gwyddion software.

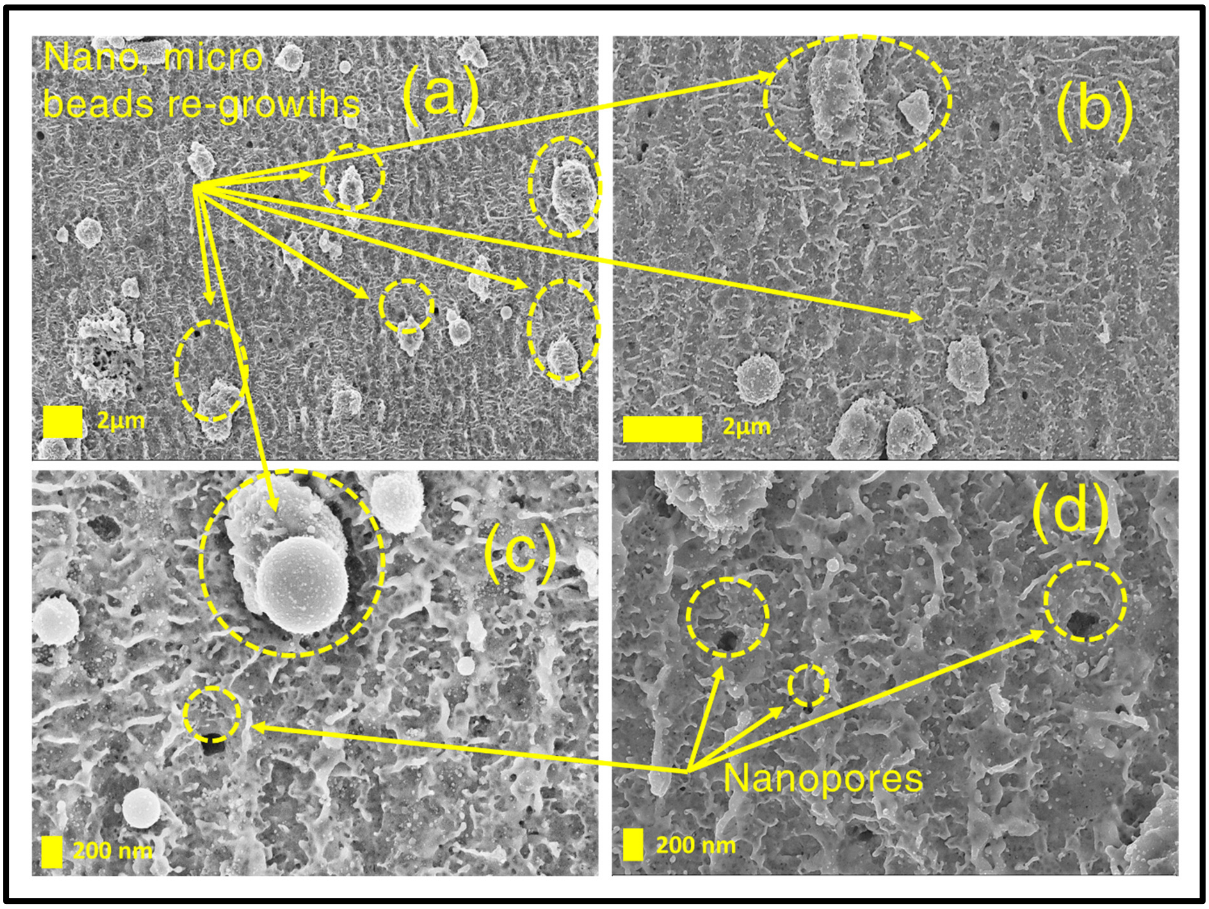

Figure S3. Lower and mid-range magnification FESEM images from a number of different regions of the Ag NS2 nanostructures portraying the nature of nano-formations. (**a**,**b**) lower magnification images corresponding to 10×, 20×, respectively. (**c**,**d**) 50× magnification images. The highlighted portions in yellow depict the different re-growth mechanisms leading to various NSs (nanopores, micro-beads, and nano-beads)

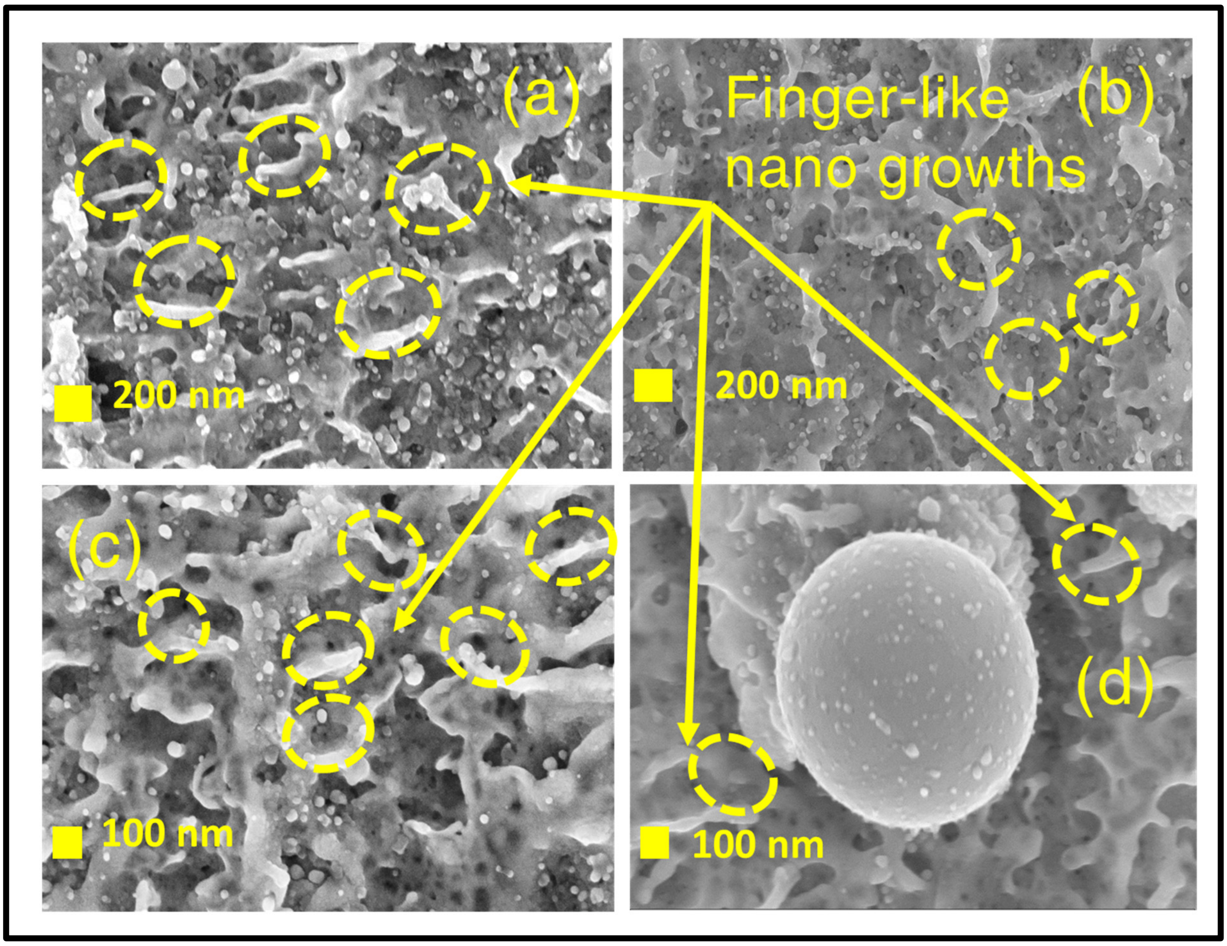

Figure S4. Higher magnification FESEM images from a number of spots in Ag NS2 nanostructure, revealing around ~100 nm growths. (**a**,**b**) 100× magnification images from various spots. (**c**,**d**) 150× magnification images from various spots. These images display the formation of finger-like nanoentities. Yellow dashed circles illustrate the finger like nanostructures on the surface post ablation.

Surface roughness analysis and EDX measurements:

We illustrate the same R.M.S. roughness variation of the Ag NSs estimated with the Gwyddion software (obtained from the FESEM images). The roughness obtained from the FESEM images and Gwyddion software is in arb. units only and we have not used this data for any quantification studies. Those were used for qualitative comparison purposes only. The root mean squared (R.M.S) roughness corresponds to a small region of the cross-raster scan fabricated final nanostructure, captured in FESEM,10 KX magnification and presented in figure 3 (a1, b1, c1, d1). To estimate the surface roughness of the various energy-dependent NSs, the afore-mentioned FESEM images were fed to Gwyddion software [http://gwyddion.net]. The 3-D surface roughness illustrations, as outputs from Gwyddion software, are presented in figures S5 [(a1) -(d1)]. The R.M.S roughness values turned out to be 23.9, 40.9, 17 and 12.3 (in arbitrary units) corresponding to AgNS1, AgNS2, AgNS3, AgNS4 respectively. The R.M.S roughness analysis in Gwyddion software is performed based on C-language based thorough statistical analysis. The moment-based quantities are expressed using integrals of the height distribution function with some powers of height. Further detailed algorithms, mentioned

in the user-guide of Gwyddion software is beyond the scope of this discussion. Subsequently, The absence of any oxidization effect in all the NSs effect has been confirmed and presented in EDX data in figures S5(a2)-(d2).

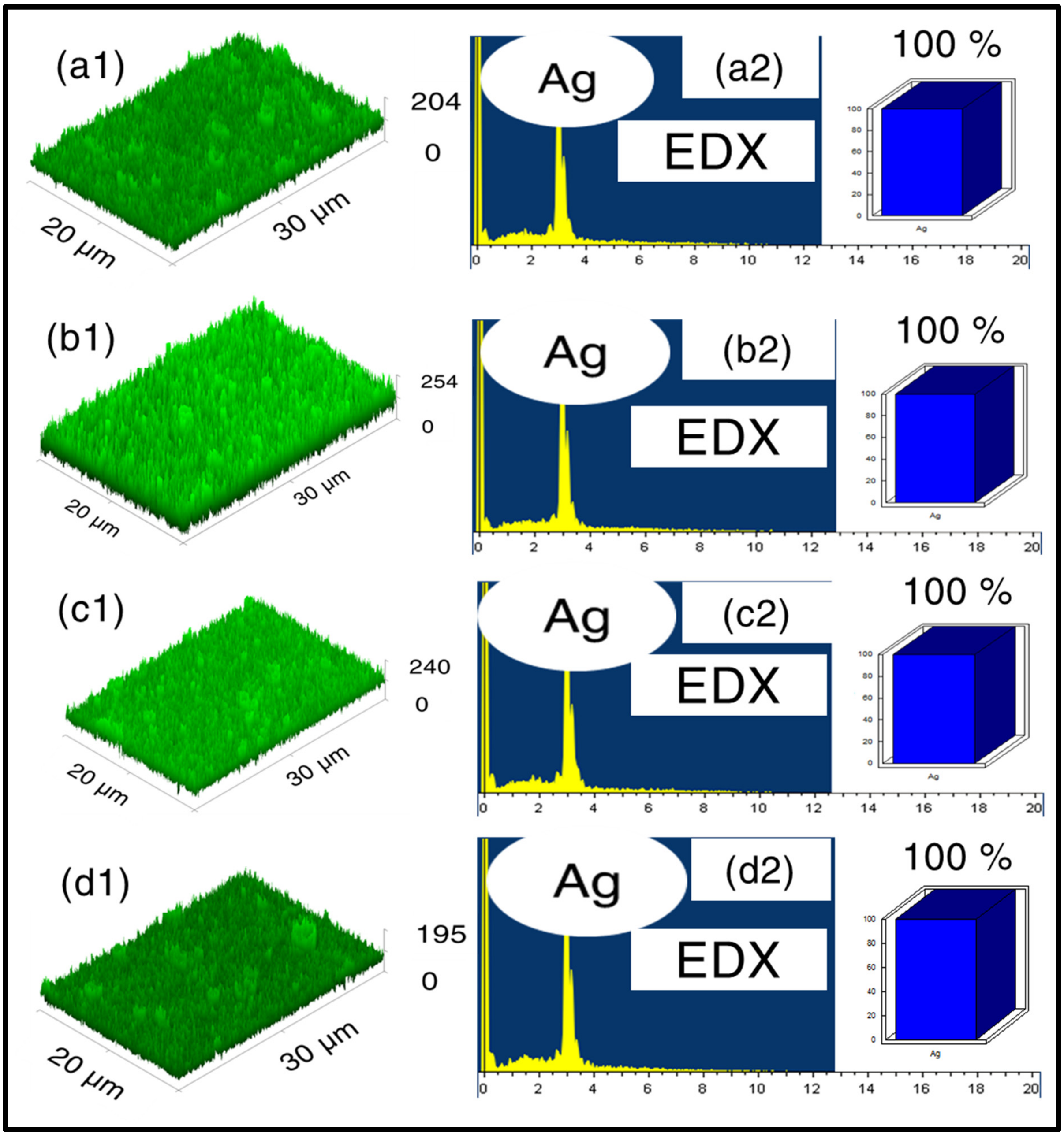

Figure S5 (**a1**,**b1**,**c1**,**d1**) display the 3D surface roughness plots from Gwyddion software. EDX spectra is exhibited in figures (**a2**,**b2**,**c2**,**d2**) corresponding to the previous order of NSs, confirming no effect of oxidation due to an appropriate preservation technique.

AFM studies:

To get an insight into the nanoscale-roughness information, the aforementioned NSs were further probed with the atomic force microscopy (AFM) technique [HITACHI AFM 5000- SPA400]. The surfaces of all the four NSs, AgNS1,

AgNS2, AgNS3, and AgNS4 were scanned in non-contact mode using dynamic force microscopy (DFM). The surface roughness image was formed from the estimation of the Coulombic force between the surface and the tip-probe, which was reflected in the cantilever amplitude change. An equal span of area (typically 3.5 µm×3.5 µm) was covered at the time of scanning for all four NSs. AFM analysis software (WSxM 5.0) was utilized to analyse the AFM data for each structure and the final data is exhibited in the form of the 3D images. In figure S6, the roughness data corresponding to AgNS1 [S6(a)], AgNS2 [S6(b)], AgNS3 [S6(c)], AgNS4 [S6(d)] is presented. The assessed surface roughness data turned out to be 33 nm, 56.1 nm, 39 nm, 34 nm, corresponding to before mentioned order of the NSs. The error in the estimation of R.M.S. roughness was found to be <5%. Ag NS2 revealed a higher surface roughness definitively, keeping a good agreement with the roughness analysis involving larger scale areas from the FESEM images.

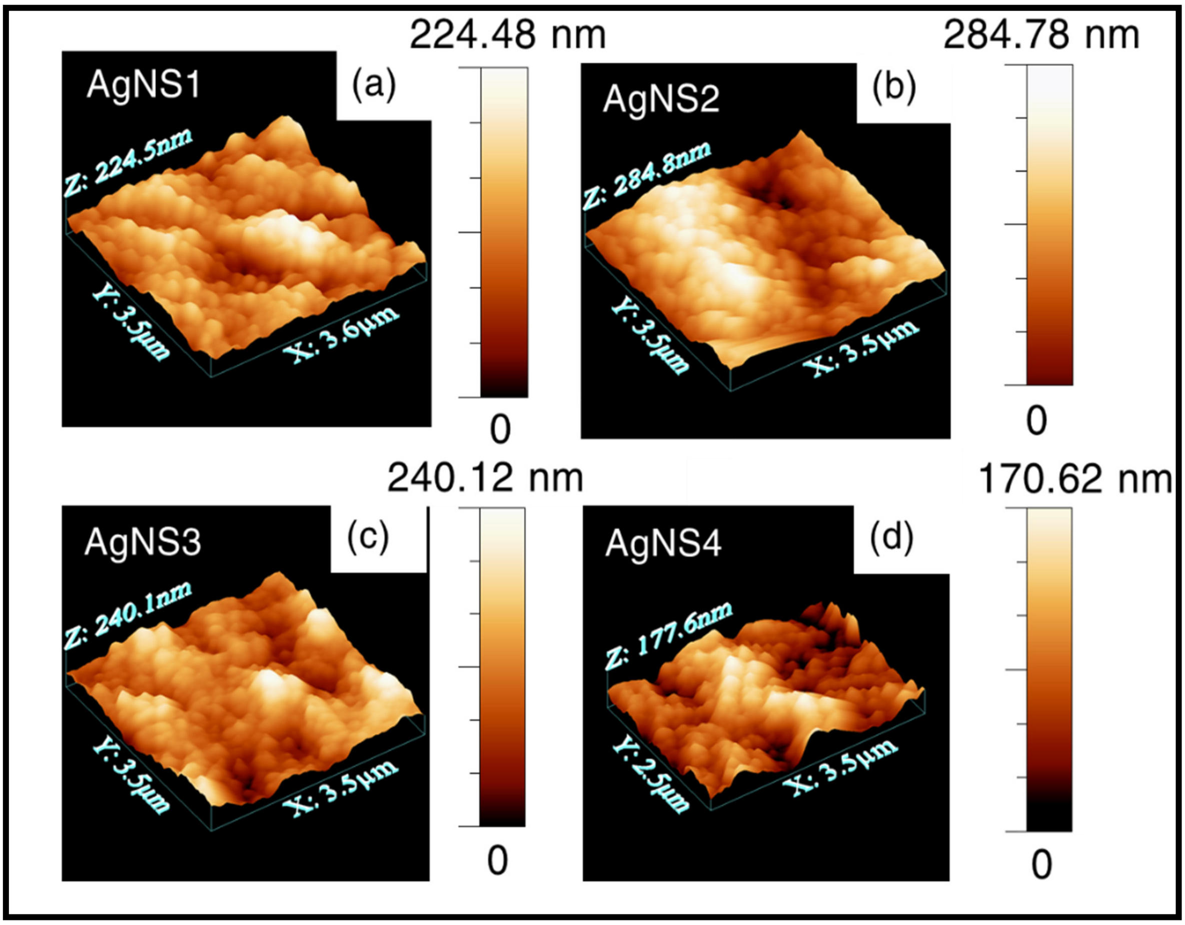

Figure S6. (**a**–**d**) provide atomic force microscopy probed 3D surface roughness profiles for AgNS1, AgNS2, AgNS3, AgNS4, respectively. Colour bars provide the depth profile at each spot.

Reflectivity studies concerned with Ag NSs:

The experimental reflectivity measurements were carried out in 10^0 and 45^0 angles of incidence over a broad band wavelength range of 250-1200 nm. In both cases, all the Ag NSs exhibited a rise in the reflectance with respect to increasing wavelength up to 1200 nm. The highest reflectance, (40-50%) has been observed in the case of Ag pristine, whereas AgNS4 (corresponding to 24 mJ pulse energy ablation) exhibited maximum reflectance of ~20% (10^0) or more

(45⁰). Both NSs- AgNS3(20 mJ), AgNS4 (12 mJ) have been observed to reveal similar reflective behaviour (around 10%), although AgNS2, formed with 16 mJ pulse energy, unveiled significantly lower reflectance characteristic response. Such outcome can be elucidated as due to the formation of higher surface roughness, offering much lower reflectivity and also a good agreement with the FESEM and AFM data. The fall in the reflectance spectra in the vicinity of 320 nm turns out to be due to higher absorption in that region. This completely matches with the similar response of the Ag substrates (fabricated using different methods) mentioned in the previous studies [2-3].

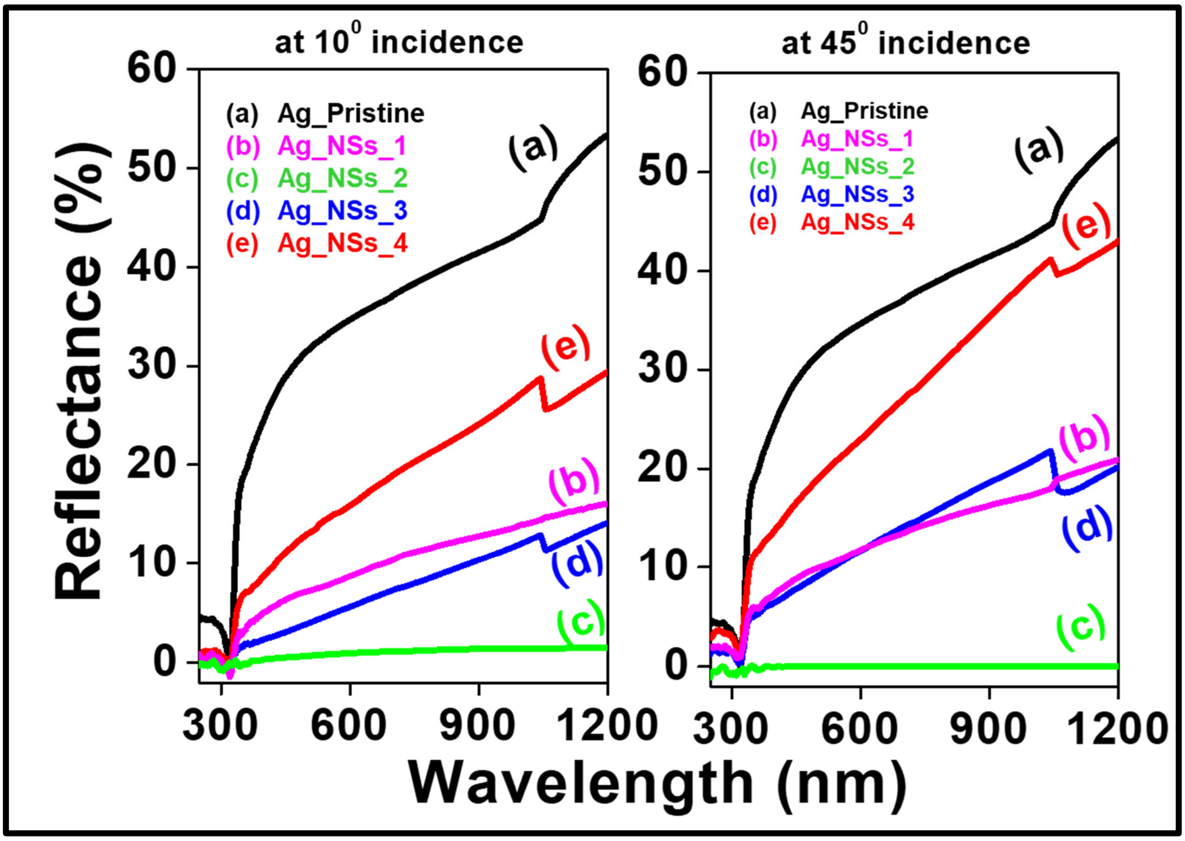

Figure S7 Reflectivity spectra for 10^0 (**left**), 45^0 (**right**) angles of incidence respectively. In both spectra, the wavelength dependent reflectivity data is represented for (a)-Ag Pristine, (b)-AgNS1, (c)- AgNS2, (d)- AgNS3, (e)- AgNS4 respectively. Due to instrumental grating change, step-like nature is found in some of the reflectance data curve (near 1050 nm).

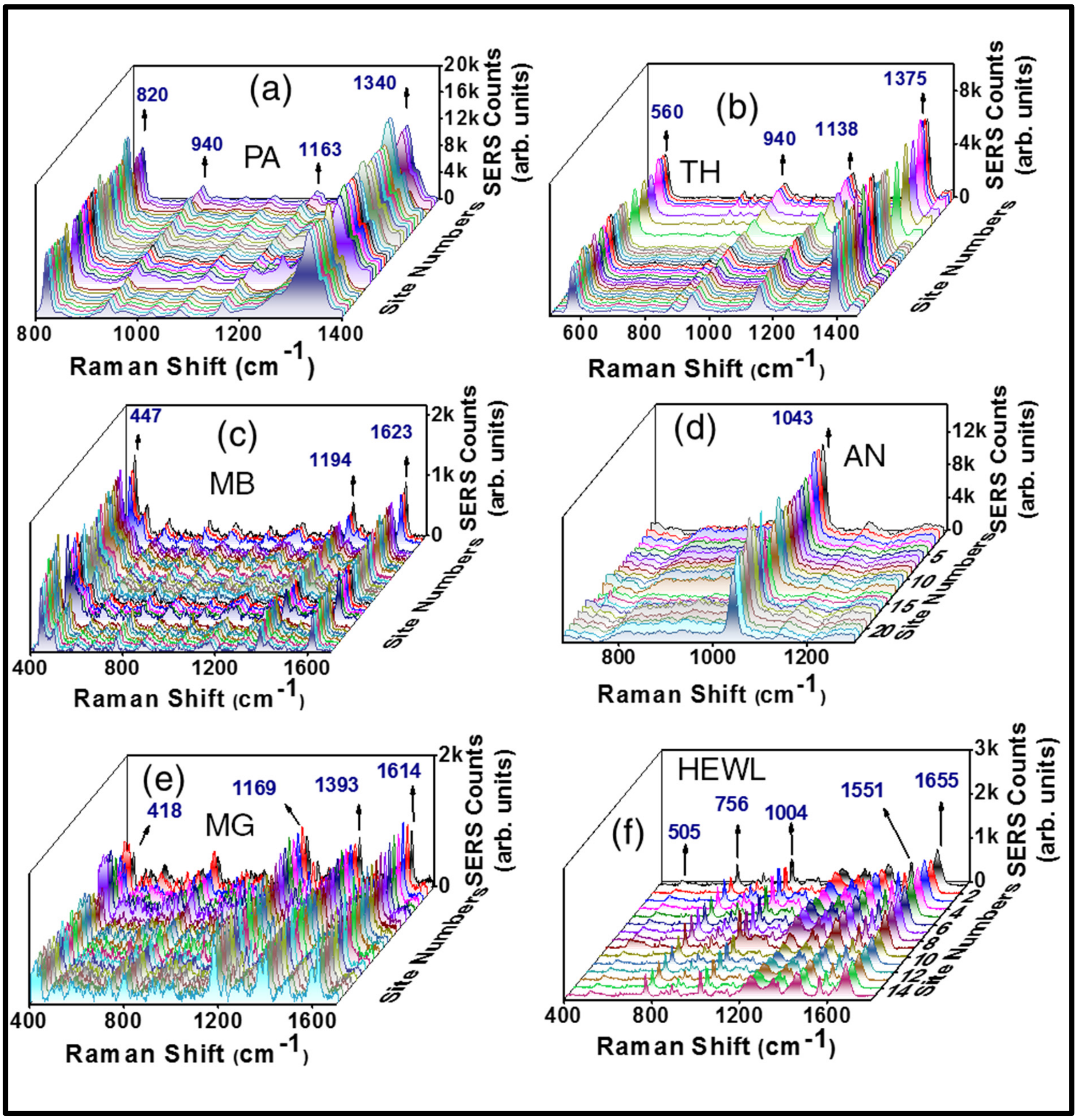

Figure S8 SERS reproducibility obtained with six different molecules using portable Raman as well as micro-Raman instrument. Portable Raman spectra were collected with 785 nm, 102 mW (30% of full power) excitation power, 5 s of accumulation time, number of spectral averaging 3 and the laser spot size ~ 100µm. Figures (a) PA, (b) TH, (c) MB, (d) AN, (e) MG illustrate the reproducibility spectra, collected from >20 random spots, for the same order of analytes. Similarly, figure (f) depicts about reproducibility spectra of HEWL (lysozyme protein biomolecule), respectively, collected with 25% of the full power (540 mW) of a

532 nm excitation laser engaged micro-Raman, using an acquisition time of 5 s, accumulation number of 3.

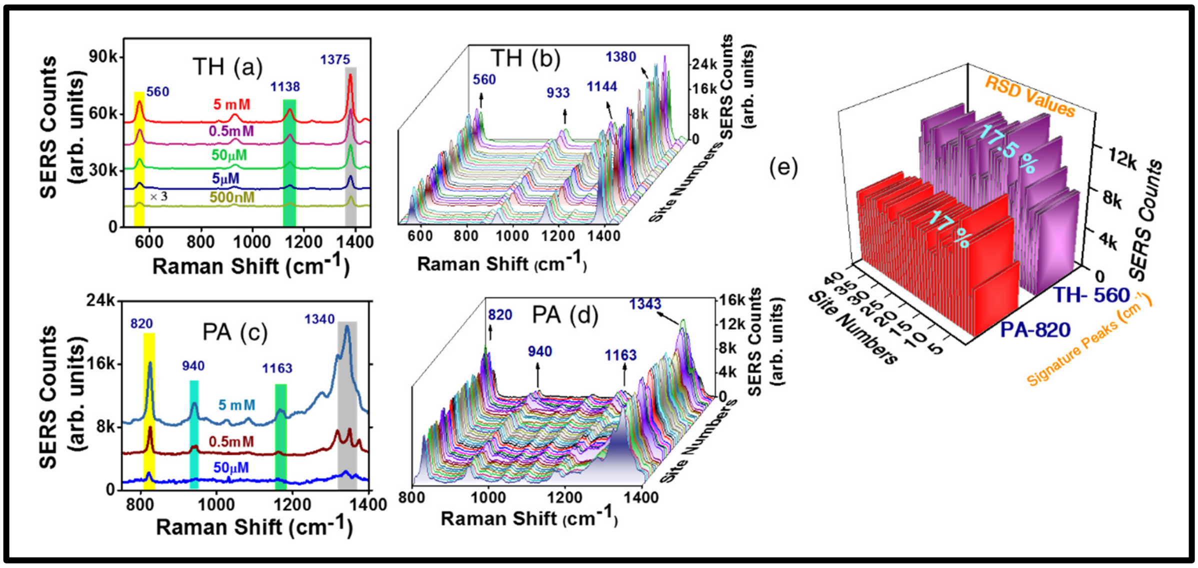

Figure S9. SERS data attained involving AgNS1, with two different molecules using portable Raman spectrometer. Portable Raman spectra were collected with 785 nm, 102 mW (30% of full power) excitation power, 5 s of accumulation time, number of spectral averaging 3. The concentration dependent Raman spectra are demonstrated for (**a**) TH (500 nM) and (**c**) PA (50 μM) whereas reproducibility spectra for >30 random spots are displayed in (**b**) TH (5 mM) and (**d**) PA (5 mM). RSD data for major peaks of both molecules (~17%) is presented in panel (**e**).

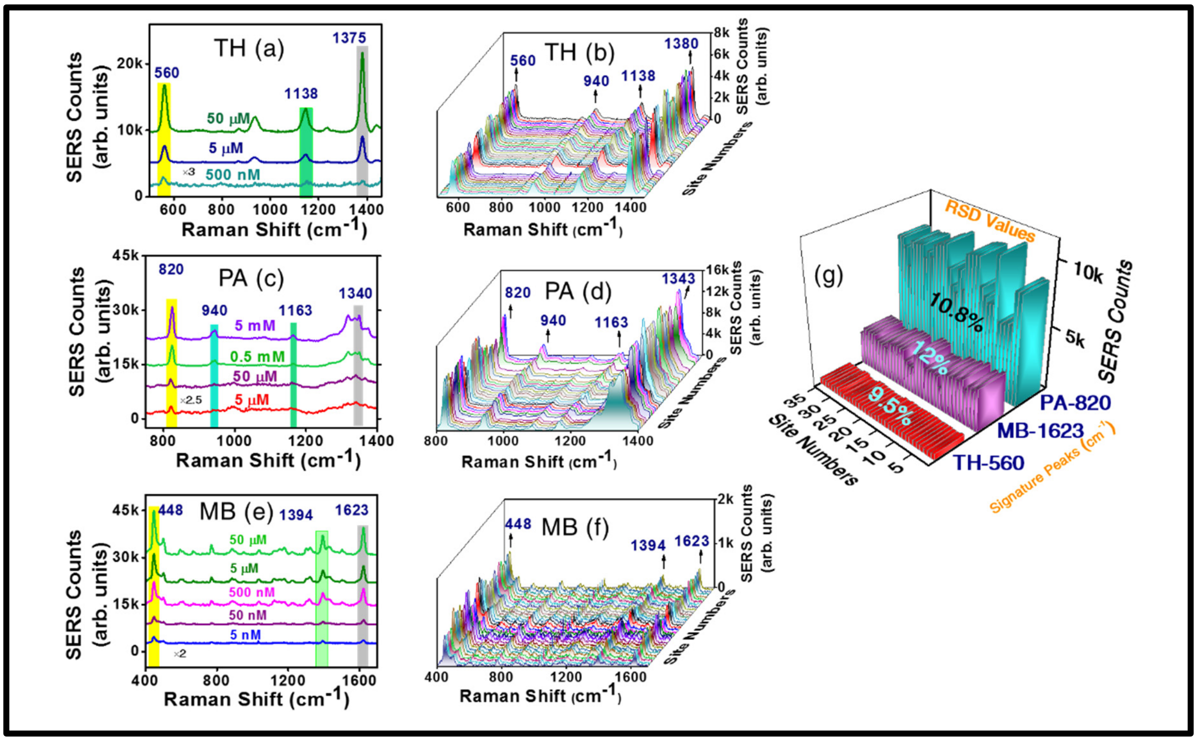

Figure S10. SERS data from AgNS3 with three different molecules using a portable Raman spectrometer. Experimental parameters were same as those mentioned in figure S9. The concentration-dependent SERS spectra are presented for (**a**) TH (500 nM), (**c**) PA (5 μM), (**e**) MB (5 nM), whereas reproducibility spectra (collected from >30 random spots) are shown in (**b**) TH (5 μM), (**d**) PA (5 mM), (**f**) MB (5 nM). RSD data for major peaks of all three molecules (<12%) has been shown in figure (**g**).

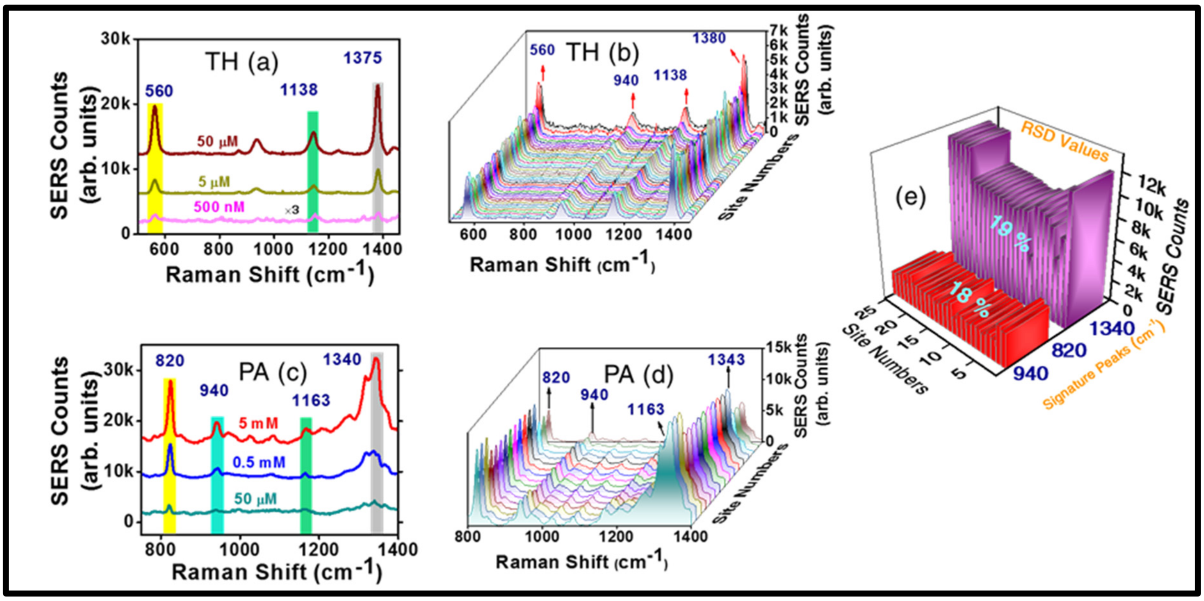

Figure S11. SERS data obtained from AgNS4 with two different molecules using a portable Raman spectrometer with the same parameters mentioned in earlier figures. The concentration-dependent Raman spectra are presented for (**a**) TH (500 nM) (**c**) PA (50 µM), and the reproducibility spectra (Obtained from >30 random spots) are presented in (**b**) TH (5 µM) (**d**) PA (5 mM). The RSD data (~18%) for major peaks of both molecules is exhibited in the panel (**e**).

Enhancement factor (EF) Calculation:

To estimate the enhancement factors (EF), the well-established mathematical relation, i.e.,

$$E.F = \frac{I_{SERS}}{I_{Raman}} \times \frac{C_{HC}}{C_{LC}}$$

where, I_{SERS} is the intensity of corresponding prominent Raman mode of analyte attained from the SERS substrate (Bessel beam structured Ag NS), I_{Raman} is the intensity of same Raman mode of analyte obtained from plain Ag substrate, C_{LC} is the lower concentration of the analyte adsorbed on the SERS substrate (BB structured Ag NS), C_{HC} is the analyte high concentration adsorbed on the plain Ag substrate.

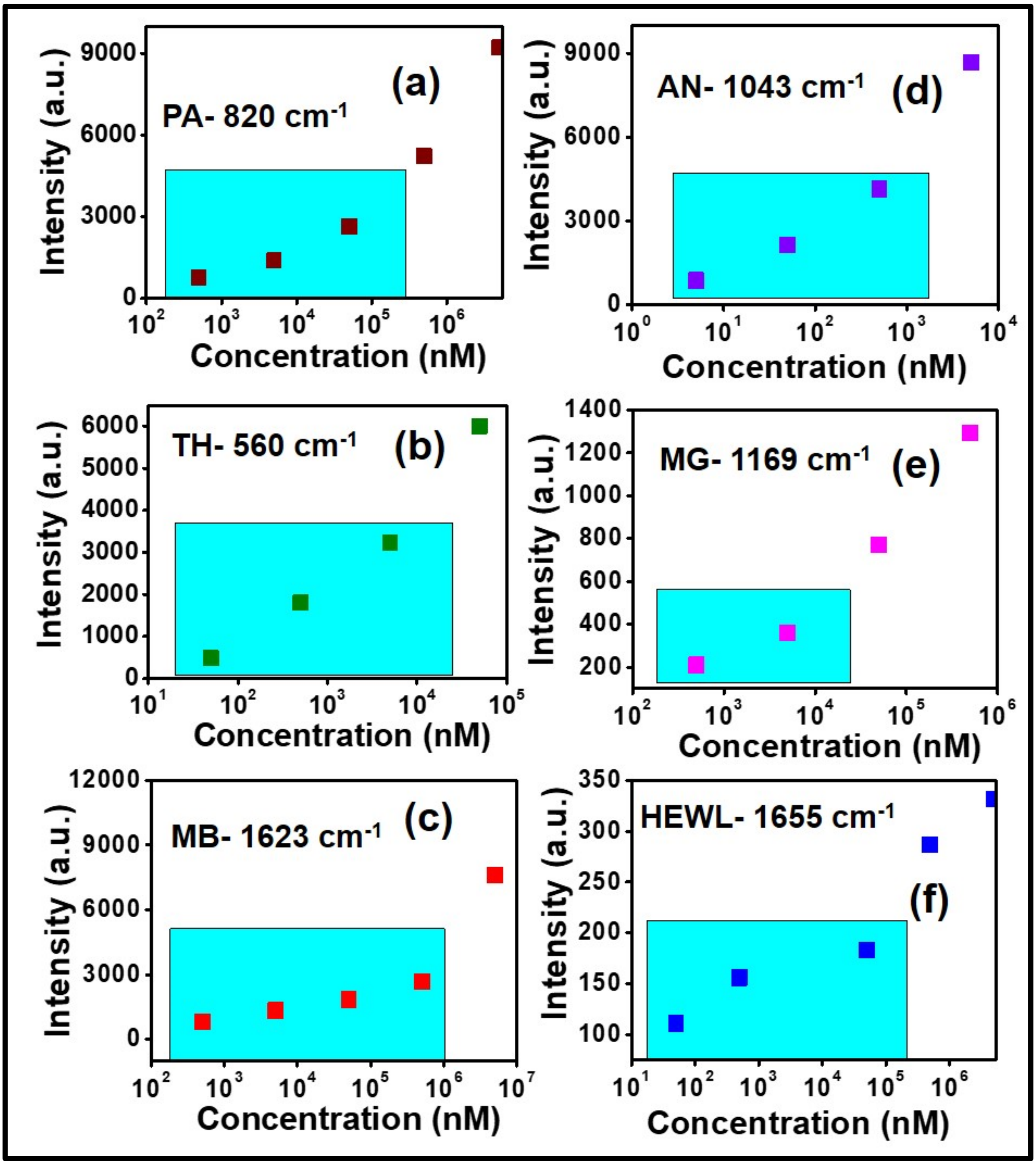

Figure S12. SERS intensity variation with analyte concentration, clearly identifying the linear nature at the lower concentration region for limit of detection (LoD) calculations. The data is demonstrated for all the analyte molecules as (**a**) PA (**b**) TH (**c**) MB (**d**) AN (**e**) MG (**f**) HEWL.

Excitation intensity dependent plasmonic response of the Ag NS:

Upon the incidence of excitation photons, the development of hotspot-localised electromagnetic enhancement due to giant optical evanescent field is the predominant impetus for the achievement of enhanced Raman signature. Trapping of optical energies concentrated on nanoscale plasmonic systems, due to surface plasmon modes, gives rise to fantastic optical phenomenon such as the SERS. To explore the induction of increasing excitation energy on the ps BB fabricated plasmonic Ag NS, the SERS experiment was performed with single dye molecule NB – Nile Blue (5 µM, $C_{20}H_{20}ClN_{3}O$), allowing the 785 nm laser excitation power to rise from 10% to 100% of the total power (340 mW). The increase in SERS counts of the signature Raman peak (590 cm^{-1}) is presented in 3D manner in figure S13(a) along with a contour plot offering a colour-representation [figure S13(b)] of the rise in SERS signal within the vicinity of the Raman peak of interest. The plot of the rise in counts was fitted to exponential function [figure S13(c)], unfolding the nature of the rise in plasmonic response from these nanoscale entities.

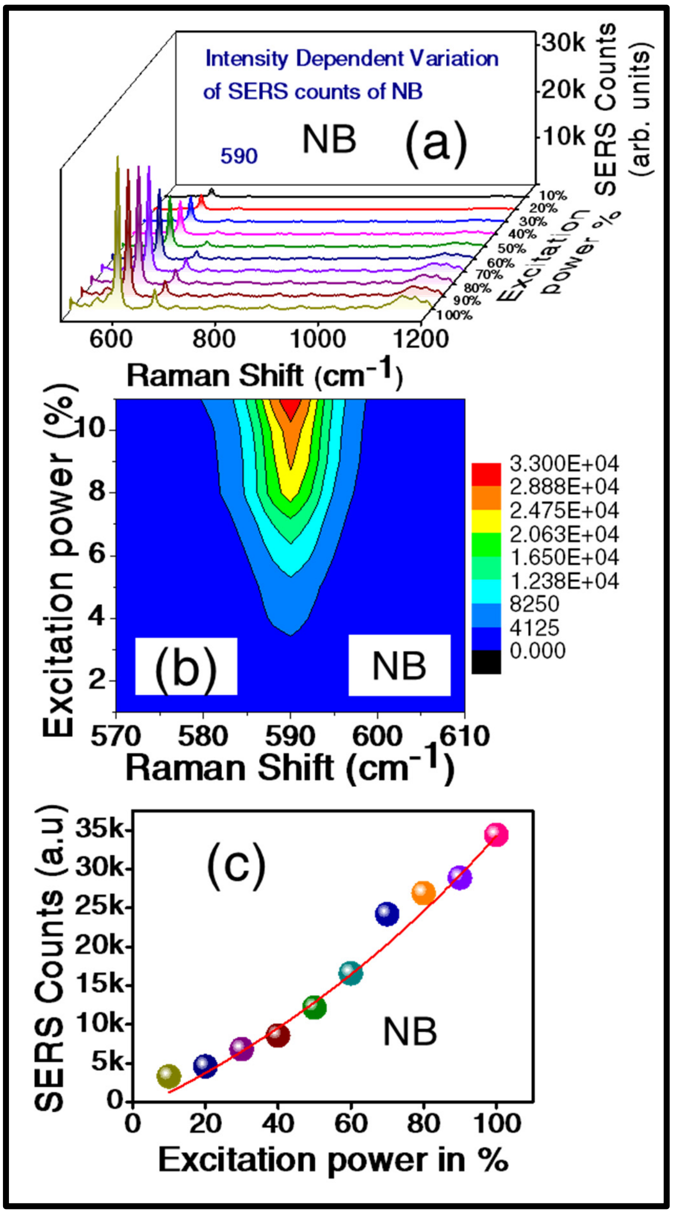

Figure S13 (a) Excitation intensity dependent Raman spectra collected by increasing incident laser power from 10-90% of the full laser power (b) Contour plot signifying the colour representation of the prominent increase in Raman signal (c) Exponential rise in the SERS counts corresponding to the 590 cm^{-1} peak of NB.

Wavelength dependent SERS studies:

The AgNS2 substrate was again employed in the wavelength dependent SERS study. An extensive Raman mapping was performed with NB molecule (5 mM) as the analyte with a micro-Raman instrument (keeping all signal accumulation parameters consistent with Raman mapping studies in figure 8 of the main MS) involving three wavelengths of 532 nm, 633 nm, 785 nm. The 3-D SERS mapping data with different wavelength excitation has been demonstrated in figure S14 with (a) 532 nm excitation (c) 633 nm excitation (e) 785 nm excitation. The corresponding SERS spectra are also presented (side wise), following the same order of wavelength [figures S14(b), S14(d), S14(f)]. The average counts, (assessed from the SERS spectra using the 590 cm^{-1} signature peak) corresponding to each excitation wavelength revealed a clear contrast. Tuning the incident laser for 532 nm (2.33 eV) excitation gave rise to ~11 times higher enhancement in the SERS signal, compared to 785 (1.57 eV) nm excited SERS counts whereas we observed a moderate increment (with ~332 average signal counts) in the case of 633 nm (1.96 eV) excitation. Furthermore, the corresponding RSDs have been estimated for each wavelength in accordance with larger area Raman map, along with smaller area responses (smaller area data is shown in figure 14 as insets). The average counts were chosen to be an appropriate statistical parameter providing a clear comparative assessment among all three batches of the SERS spectra. We have evidently observed a distinct fall in the average counts following the increase in Raman excitation wavelength. The gradual increase in the excitation profile, as the excitation wavelength (λ) becomes shorter, can be attributed to the λ^{-4} dependence of scattering nature of the Raman signal [5].

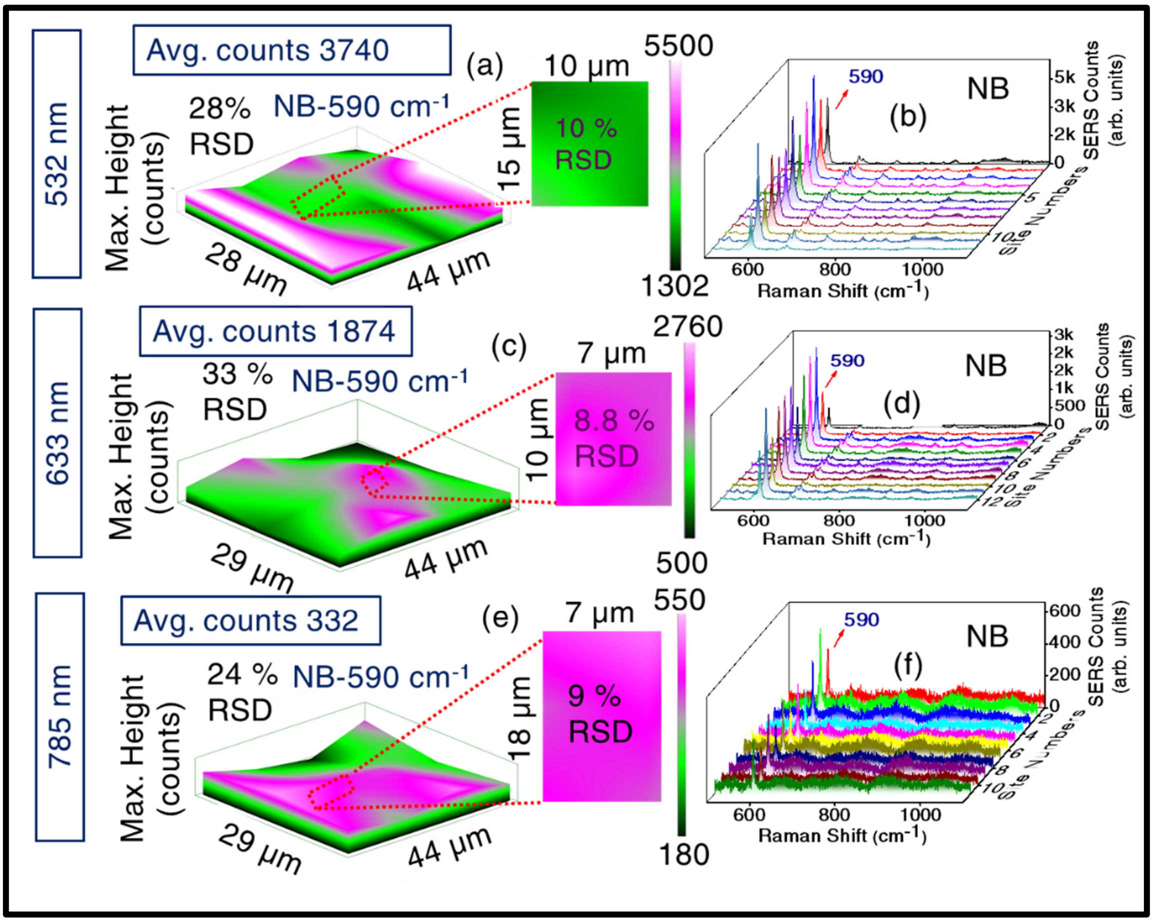

Figure S14 Wavelength dependent Raman experiment performed with micro-Raman detecting the NB molecule, keeping other experimental parameters exactly unchanged from the aforementioned study at figure 8. 3D Raman mapping data of NB is presented corresponding to (**a**) 532 nm (**c**) 633 nm (**e**) 785 nm excitation. The corresponding SERS spectra generated while mapping is presented in (**b**) 532 nm (**d**) 633 nm (**f**) 785 nm. Corresponding whole-some RSD estimations are also provided, along with smaller area RSD data extracted from cursor spectrum feature in LabRAM mapping software. Average counts were estimated from each Raman mapped data, clearly signifying the gradual decline in SERS counts with redshift in excitation wavelength.

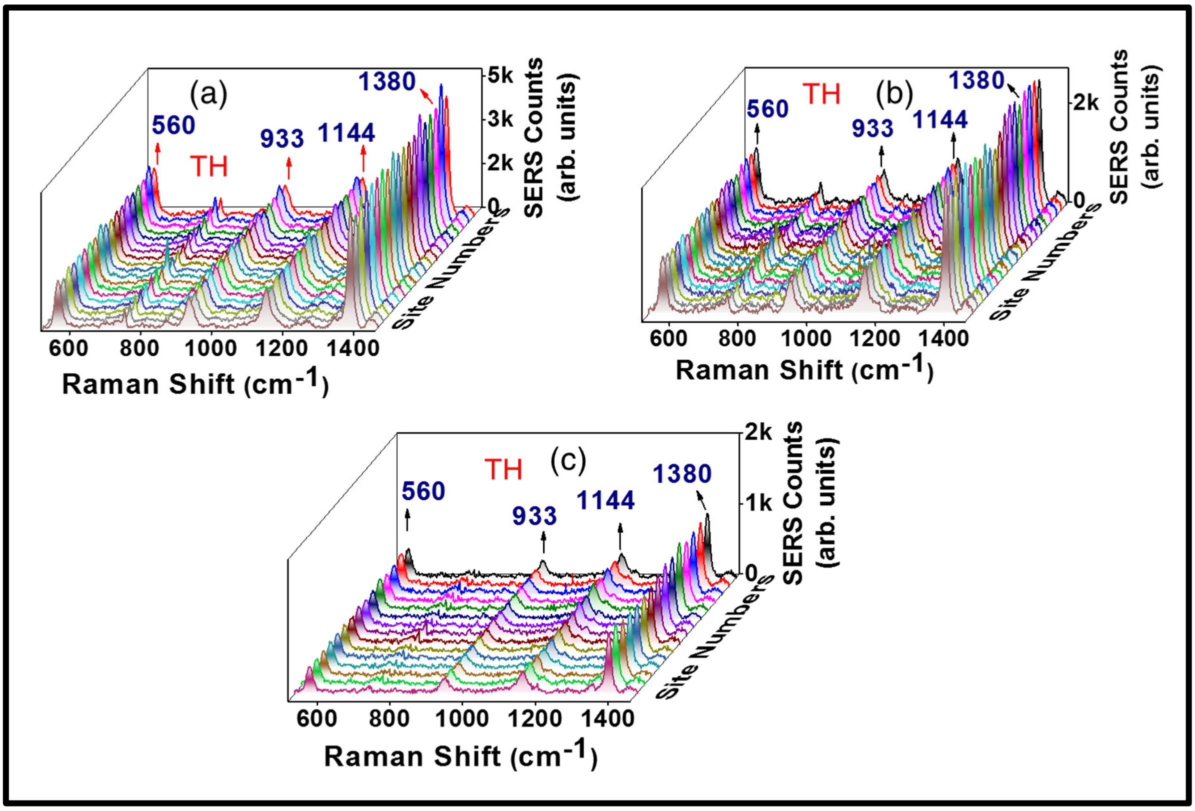

Figure S15. Reproducibility spectra for TH concerned with three Au-coated AgNS2 (a) [AgNS2 + 10 nm Au], (b) [AgNS2 + 20 nm Au], (c) [AgNS2 + 30 nm Au]. The concentrations of TH utilized has been 500 nM, 5 nM, 5nM for the three abovementioned substrates, respectively.

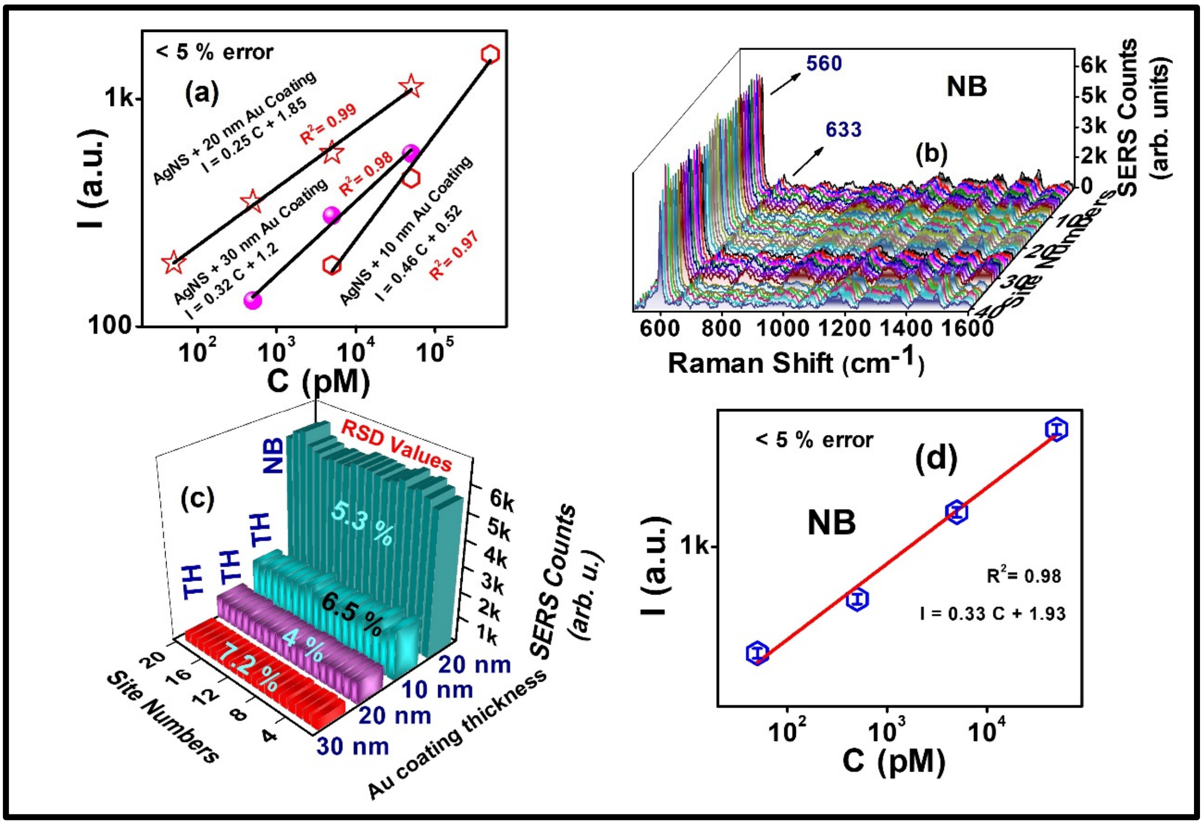

Figure S16. (a) SERS peak intensity versus concentration in the case of TH (560 cm^{-1} peak) involving all the Au-coated substrates (b) Reproducibility spectra of NB (50 nM; 590 cm^{-1} peak) collected with portable Raman engaging 20 nm Au coated AgNS2 (c) RSD bar graph representations TH as analyte involving all three 10 nm, 20 nm, 30 nm Au coated SERS substrates, along with the RSD data for NB acquired employing particularly 20 nm Au coated AgNS2 (d) Intensity versus concentration dependence in NB.

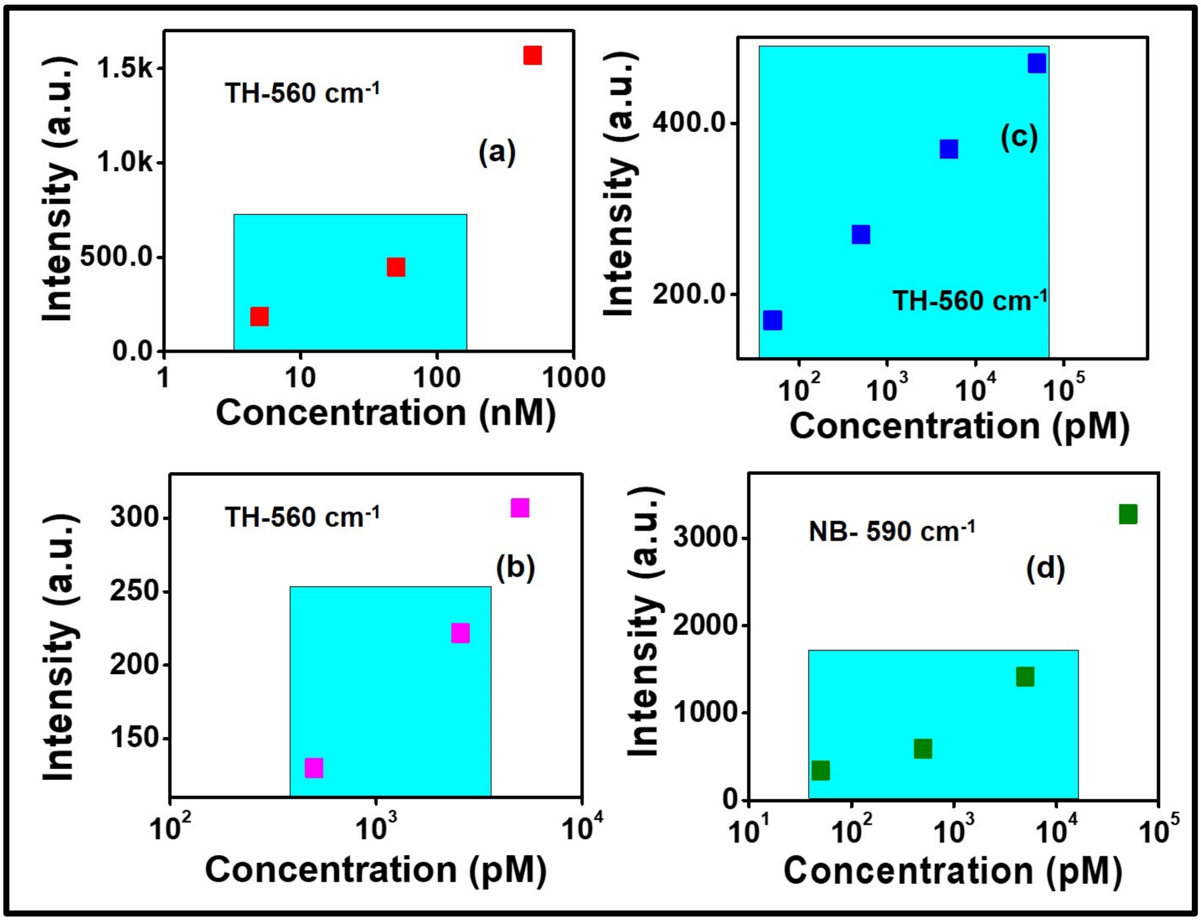

Figure S17. SERS intensity variation with analyte concentration, clearly denoting the linear nature at the lower concentration region for limit of detection (LoD) calculations. The data is demonstrated for the TH analyte molecule concerning three Au coated substrates as (a) [AgNS2 + 10 nm Au], (b) [AgNS2 + 20 nm Au], (c) [AgNS2 + 30 nm Au]. Particularly, the same for NB molecule (d) SERS data collected with [AgNS2 + 20 nm Au] substrate is also presented.

Model calculation for the estimation of EF:

In case of the detection of Thiram (TH), utilizing (AgNS2 +20 nm Au coating) as SERS substrate,

$$I_{SERS} = 222\ counts,\ I_{Raman} = 220,\ C_{LC} = 50\ pM,\ C_{HC} = 5\ mM$$

And, hence, the EF is now,

$$E.F. = \frac{222}{200} \times \frac{50 \times 10^{-12}}{5 \times 10^{-3}} = 1.01 \times 10^{8}$$

The corresponding Raman spectra of Thiram (5 mM), taken on bare (not laser structured) silver, and SERS signal corresponding to lowest possible detected trace of Thiram (50 pM) on (AgNS2 +20 nm Au coating) as SERS substrate, has been presented on figure S18.

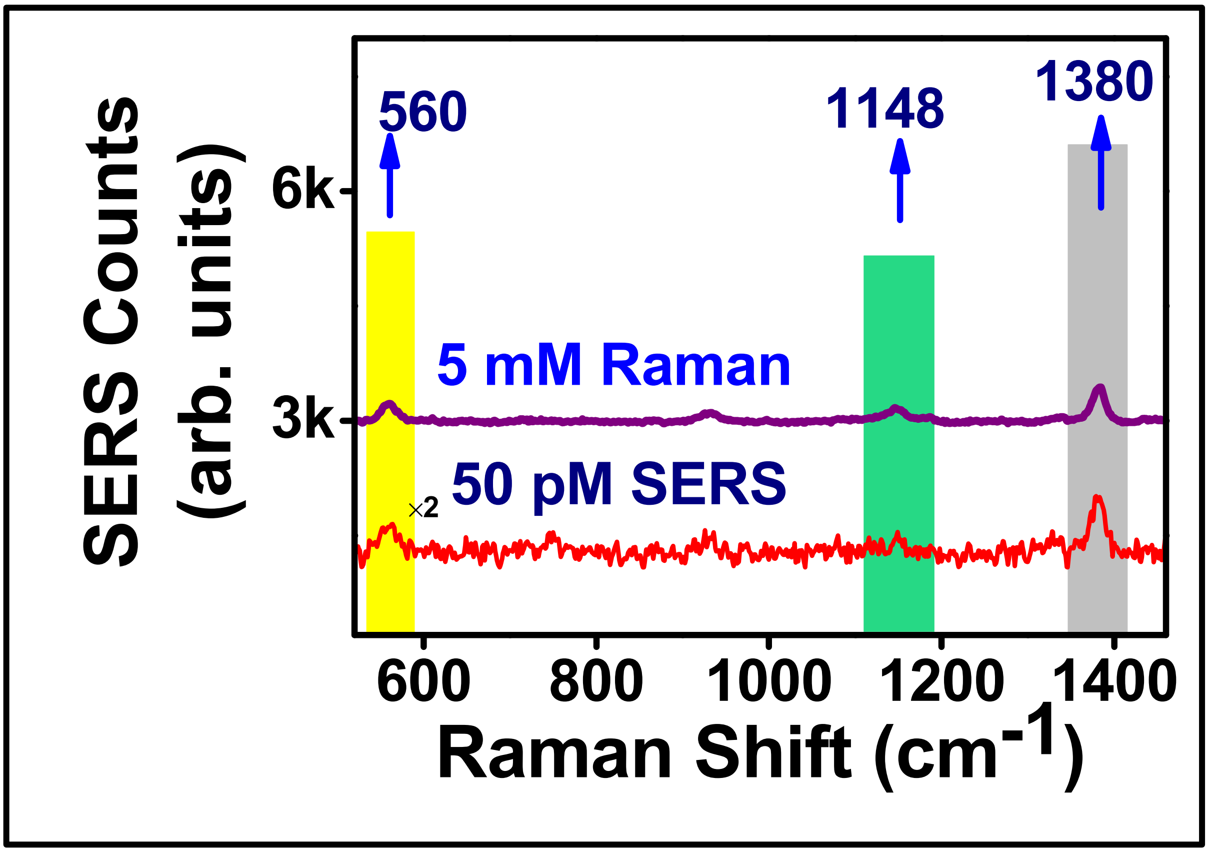

Figure S18. Raman spectra of Thiram (5 mM, in purple), taken on bare (not laser structured) silver, and SERS signal corresponding to lowest possible detected trace of Thiram (50 pM, in red) on (AgNS2 +20 nm Au coating) as SERS substrate. The detection is performed with portable Raman instrument i-Raman plus. Acquisition parameters are mentioned in main manuscript.

Typical LOD calculation:

For PA, $\frac{3\sigma}{b} = \frac{3 \times 32}{0.27\ (^{a.u}/_{nM})} = 360\ nM$

where standard deviation of SERS intensity at lowest concentration; σ = 32, value of b = 0.27, taken from figure 7(a), linear plot.

Table S1. Sensing performance comparison of the recently reported SERS substrates with those reported in the present work.

Serial No.	SERS Substrate	Fabrication technique	Lowest Analyte concentration traced	Enhancement Factor	Reference (in main MS)
1	Ag/ Au NPs multilayer Al_2O_3 film (MLF) hybrid system	Thermal evaporation, displacement reaction	Rhodamine 6G dye, 10^{-10} M	10^6	91
2	PDMS plano-convex microlens SERS-active substrate in combination with an Au film	liquid–liquid interface self-assembly method followed by fine treatment	Rhodamine 6G dye, 10^{-9} M	-	90
3	plasmonic nanoparticles embedded in a scaffold of nano-porous silicon	Chemical synthesis with $HAuCl_4$	Methylene blue dye, 10^{-12} M	10^9	71
4	Metal NPs/FEP films	Femtosecond LIPAA technique	Thiram (0.1 ppm, 7.96 ng/ cm^2)	10^7	61
5	Hierarchically Assembled Plasmonic Metal-Dielectric-Metal Hybrid Nano-Architectures	Thermal evaporation followed by the thermal annealing and atomic layer deposition (ALD) of the Al_2O_3 dielectric layer	Rhodamine 6G dye, 10^{-11} M	10^8	76
6	ZnO/Ag Core-Satellite Nanostructures	Chemical synthesis involving pyrolysis method	Rhodamine 6G dye, 10^{-13} M	10^8	74

7	Bessel beam structured Ag substrate	picosecond laser ablation	TH (50 nM)<br>PA 500 nM<br>MB500 pM<br>AN5 μM<br>MG500 pM	highest 10^8	This work

References

1. Wu, P.; Sui, C.; Huang, W. Theoretical Analysis of a Quasi-Bessel Beam for Laser Ablation. *Photonics Res.* **2014**, *2*, 82.
2. Vergöhl, M.; Malkomes, N.; Szyszka, B.; Neumann, F.; Matthée, T.; Bräuer, G. Optimization of the Reflectivity of Magnetron Sputter Deposited Silver Films. *J. Vac. Sci. Technol. A Vacuum, Surfaces, Film.* **2000**, *18*, 1632–1637.
3. Chen, D.; Zhang, Y.; Bessho, T.; Kudo, T.; Sang, J.; Hirahara, H.; Mori, K.; Kang, Z. Formation of Reflective and Conductive Silver Film on ABS Surface via Covalent Grafting and Solution Spray. *Appl. Surf. Sci.* **2015**, *349*, 503–509.
4. Schulz, L. G. The Optical Constants of Silver, Gold, Copper, and Aluminum I The Absorption Coefficient K. *J. Opt. Soc. Am.* **1954**, *44*, 357.
5. Jaculbia, R. B.; Imada, H.; Miwa, K.; Iwasa, T.; Takenaka, M.; Yang, B.; Kazuma, E.; Hayazawa, N.; Taketsugu, T.; Kim, Y. Single-Molecule Resonance Raman Effect in a Plasmonic Nanocavity. *Nat. Nanotechnol.* **2020**, *15*, 105–110.
